# The circadian clock remains intact, but with dampened hormonal output in heart failure

**DOI:** 10.1016/j.ebiom.2023.104556

**Published:** 2023-04-17

**Authors:** Sandra Crnko, Markella I. Printezi, Peter-Paul M. Zwetsloot, Laurynas Leiteris, Andrew I. Lumley, Lu Zhang, Isabelle Ernens, Tijn P.J. Jansen, Lilian Homsma, Dries Feyen, Martijn van Faassen, Bastiaan C. du Pré, Carlo A.J.M. Gaillard, Hans Kemperman, Marish I.F.J. Oerlemans, Pieter A.F.M. Doevendans, Anne M. May, Nicolaas P.A. Zuithoff, Joost P.G. Sluijter, Yvan Devaux, Linda W. van Laake

**Affiliations:** a Department of Cardiology, Experimental Cardiology Laboratory, University Medical Centre Utrecht, Utrecht, the Netherlands; b Regenerative Medicine Centre, Circulatory Health Laboratory, University Medical Centre Utrecht, Utrecht, the Netherlands; c Cardiovascular Research Unit, Luxembourg Institute of Health, Luxembourg; d Department of Cardiology, Radboud University Medical Centre, Nijmegen, the Netherlands; e Department of Internal Medicine, Jeroen Bosch Hospital, ‘s-Hertogenbosch, the Netherlands; f Department of Medicine and Cardiovascular Institute, Stanford University, Stanford, CA, USA; g Department of Laboratory Medicine, University Medical Centre Groningen, University of Groningen, the Netherlands; h Division of Internal Medicine, Erasmus Medical Centre, Rotterdam, the Netherlands; i Division of Internal Medicine and Dermatology, University Medical Centre Utrecht, Utrecht, the Netherlands; j Central Diagnostic Laboratory, University Medical Centre Utrecht, Utrecht, the Netherlands; k Netherlands Heart Institute, Utrecht, the Netherlands; l Central Military Hospital, Utrecht, the Netherlands; m Department of Epidemiology, Julius Centre for Health Sciences and Primary Care, University Medical Centre Utrecht, Utrecht University, Utrecht, Netherlands; n Department of Data Science and Biostatistics, Julius Centre for Health Sciences and Primary Care, University Medical Centre Utrecht, Utrecht University, Utrecht, Netherlands; o Utrecht University, Utrecht, the Netherlands

**Keywords:** Human heart failure, Circadian rhythms, Zebrafish, Mouse, Melatonin, Cortisol

## Abstract

**Background:**

Circadian (24-h) rhythms are important regulators in physiology and disease, but systemic disease may disrupt circadian rhythmicity. Heart failure (HF) is a systemic disease affecting hormonal regulation. We investigate whether HF affects the rhythmic expression of melatonin and cortisol, main endocrine products of the central clock, and cardiac-specific troponin in patients. We corroborate the functionality of the peripheral clock directly in the organs of translational models, inaccessible in human participants.

**Methods:**

We included 46 HF patients (71.7% male, median age of 60 years, NYHA class II (32.6%) or III (67.4%), ischemic cardiomyopathy (43.5%), comorbidities: diabetes 21.7%, atrial fibrillation 30.4%), and 24 matched controls. Blood was collected at seven time-points during a 24-h period (totalling 320 HF and 167 control samples) for melatonin, cortisol, and cardiac troponin T (cTnT) measurements after which circadian rhythms were assessed through cosinor analyses, both on the individual and the group level. Next, we analysed peripheral circadian clock functionality using cosinor analysis in male animal HF models: nocturnal mice and diurnal zebrafish, based on expression of core clock genes in heart, kidneys, and liver, every 4 h during a 24-h period in a light/darkness synchronised environment.

**Findings:**

Melatonin and cortisol concentrations followed a physiological 24-h pattern in both patients and controls. For melatonin, acrophase occurred during the night for both groups, with significantly decreased amplitude (median 5.2 vs 8.8, *P* = 0.0001) and circadian variation ([maximum]/[minimum]) in heart failure patients. For cortisol, mesor showed a significant increase for HF patients (mean 331.9 vs 275.1, *P* = 0.017) with a difference of 56.8 (95% CI 10.3–103.3) again resulting in a relatively lower variation: median 3.9 vs 6.3 (*P* = 0.0058). A nocturnal blood pressure dip was absent in 77.8% of HF patients.

Clock gene expression profiles (*Bmal, Clock*, *Per*, *Cry*) were similar and with expected phase relations in animal HF models and controls, demonstrating preserved peripheral clock functionality in HF. Furthermore, oscillations in diurnal zebrafish were expectedly in opposite phases to those of nocturnal mice. Concordantly, cTnT concentrations in HF patients revealed significant circadian oscillations.

**Interpretation:**

Central clock output is dampened in HF patients while the molecular peripheral clock, as confirmed in animal models, remains intact. This emphasises the importance of taking timing into account in research and therapy for HF, setting the stage for another dimension of diagnostic, prognostic and therapeutic approaches.

**Funding:**

10.13039/501100002996Hartstichting.

**Research in context**

**Evidence before this study**

Heart failure is a leading cause of death worldwide, with limited treatment options and poor prognosis. Heart failure is a systemic disease affecting hormonal regulation. Circadian (24-h) rhythms are known to have an important role in the cardiovascular system and in hormonal regulation, however whether the circadian clock remains functional in the failing heart is unknown and difficult to study. However, neurohormones relevant in heart failure have day/night rhythms; patients with heart failure often suffer from insomnia and, vice versa, insomnia has been found to increase the risk of incident heart failure. However, in spite of the growing realisation that circadian rhythms are an important factor in heart failure, the extent of its involvement in the disease genesis, progression and treatment is yet unknown. Untangling the circadian expression profile of heart failure patients will help to better understand the relationship between circadian rhythms and heart failure, provide an understanding of the underlying pathways, and represents a potential approach to finding druggable targets.

**Added value of this study**

In the present study, we showed that the central circadian clock, as reflected by 24-h melatonin and cortisol levels, is intact but dampened in patients with heart failure. Cardiac troponin T concentrations exhibit a circadian rhythm in heart failure patients, further confirming the preservation of the cardiac circadian clock. Peripheral clocks are intact in organs of nocturnal (mouse) and diurnal (zebrafish) animal models of heart failure: heart, kidneys and liver in mice, and heart and kidneys in zebrafish. Since the circadian clock remains functional in heart failure, timing of interventions and measurements should be taken into account in clinical studies to minimise variation and maximise effect.

**Implications of all the available evidence**

The high prevalence of heart rate and blood pressure non-dippers in our heart failure population, combined with their lower circadian variation of melatonin and cortisol provides new vantage points for future treatment options for heart failure. Earlier evidence has pointed in the direction of melatonin as a possible (preventive or curative) treatment option for heart failure, but without considering the circadian clock. Our study provides support for novel clinical studies investigating the effects of timed melatonin supplementation in this domain. Furthermore, the quality of life for patients suffering from heart failure could be improved through central clock therapy by implementing various behavioural interventions such as light and activity therapy focusing on central clock output and subjective sleep quality.

## Introduction

Circadian rhythms are increasingly seen as important regulators in (patho)physiology.^1^ They are driven by a central clock located in the suprachiasmatic nucleus of the brain and peripheral clocks found in almost every tissue. The central clock synchronises peripheral clocks via neurohumoral signals (e.g. cortisol and melatonin). Additionally, peripheral clocks respond to tissue-specific synchronizers such as food intake and exercise. The cardiovascular system and its hormonal interactors are particularly sensitive to circadian regulation. Being present in each cardiovascular cell type, the molecular clock influences many cardiovascular processes, including heart rate, metabolism, signalling, contractility, and vascular tonus. Its disruption has been linked with the development and incidence of cardiovascular diseases. Acute myocardial infarction and arrhythmias peak in the morning,^1^ and a greater risk of major cardiac events has been described after morning heart surgery, compared to afternoon surgery.^2^

Heart failure (HF) is a systemic disease affecting hormonal regulation. Several observations suggest a connection between the circadian clock and heart failure (HF): relevant neurohormones display diurnal rhythms,^3^ HF patients often suffer from insomnia, and insomnia has been found to increase the risk of HF.^4^ Furthermore, circadian mechanisms play an important role in neurobiology of the murine healthy cognitive system.^5^ HF changes neuron morphology and function, potentially causing the neurocognitive impairments observed in HF patients, and the loss of the circadian mechanism alters neurobiological gene adaptations to HF. Whether HF affects the circadian clock remains unknown, but would be plausible given the massive neurohormonal activation in HF.^6^ On the other hand, the circadian clock is an evolutionarily conserved mechanism that may receive priority in remaining intact during the course of a disease.

Circadian rhythms have also proven relevant in timing therapy. Circadian oscillations of pharmacokinetics and dynamic intrinsic cellular function, may all affect the efficacy and side effects of drugs.^1^ Additionally, non-pharmacological HF interventions such as exercise, diet, and pacemaker/implantable cardioverter-defibrillator therapy may vary in their effectiveness and optimal settings according to the time of day. Importantly, HF therapy does not only affect signalling pathways in the heart, but also influences other organs (e.g. kidneys) regulated by the circadian clock.^7^

Although it is increasingly recognised that circadian rhythms are important in cardiovascular pathophysiology, their involvement in HF genesis, progression, and treatment is yet unknown. Given the potential implications for tailoring existing and developing new therapies targeting components of the circadian systems, it is important to establish whether the circadian clock is impaired following HF.

Here, we take a translational approach to characterise circadian rhythmicity in HF patients, and provide insight into molecular peripheral clock function of multiple HF-related organs in two independent animal HF models (Supplementary Fig. S1). Of note, throughout the manuscript we use the term circadian rhythms when referring to rhythms that exist with periods of 24 h, whether this occurs intrinsically or as a response to 24-h environmental changes. Although, strictly speaking, only rhythms that have been shown to persist under constant environmental conditions should be called circadian, we chose this terminology for practical purposes. The terms diurnal and nocturnal are used to depict the activity/rest periods of animals during the 24 h period: diurnal animals = active phase during the day and rest phase during the night, nocturnal animals = active phase during the night and rest phase during the day.

## Methods

### Ethics

The observational case–control study complies with the Declaration of Helsinki (64th WMA General Assembly, Fortaleza, Brazil, October 2013) and the Medical Research Involving Human Subjects Act (WMO). It was approved by the medical ethical committee of University Medical Centre Utrecht (UMCU; study number 14/471). All subjects signed informed consent. Animal experiments were carried out in accordance with the Guide for the Care and Use of Laboratory Animals and ARRIVE guidelines, with prior approval by the local Animal Ethical Experimentation Committee (Utrecht University: mice, DEC: 2014. II.03.022; Luxembourg Institute of Health: zebrafish, LRCV-2016-01).

### Study design: human heart failure

Two subject groups were included: HF with reduced ejection fraction (HFrEF) patients (NYHA II/III, euvolemic) and age- and sex-matched healthy individuals (UMCU, 2015–2019). All HF patients 18–85 years old that were admitted to the UMC Utrecht in a period from 2015 to 2019 were considered for inclusion. The following exclusion criteria were applied: blindness, end stage renal failure, fever, elevated CRP, use of sedatives within 48 h of start study, severe comorbidity possibly influencing circadian rhythms, systemic glucocorticoid use, and intravenous treatment of amiodarone, diuretics or inotropes. Sample size was based on previous studies in renal failure, diabetes or healthy controls.^8^, ^9^, ^10^, ^11^ For matching, we sorted patients based on sex and age and matched 2:1 by biological sex (male/female; in all subjects biological sex at birth was the same as sex/gender during the investigation) and age, allowing a maximum of 10% difference between cases and controls. Blood was collected from an intravenous cannula at 9 AM, 1 PM, 5 PM, 9 PM, 1 AM, 5 AM, and 9 AM, totalling 320 HF and 167 control samples. Serum melatonin and cortisol were measured by liquid chromatography-tandem mass spectrometer,^12^ and cardiac troponin T (cTnT) concentrations with Troponin T-high sensitive immunoassay (Roche Diagnostics, Indianapolis).

Chronotype and sleep quality were determined by VOA-questionnaire (Vragenlijst Ochtend/Avond typering) and Epworth Sleepiness Scale (ESS), respectively. Following hospitalisation, subjects wore a wrist actometer (Philips Respironics Actiwatch 2) for five days, while maintaining usual sleep and activity patterns.

For the HFrEF group, 24-h blood pressure (BP, mmHg) and heart rate (HR, beats per minute (BPM)) measurements were obtained.

### Study design: mouse heart failure

Male C57BL/6 mice (Jackson), aged 10–12 weeks were housed in a 12-h light/12-h dark cycle (lights-on = zeitgeber 0 (ZT0), lights-off = ZT12). Water and food were provided *ad libitum.* Myocardial infarction (MI) was induced by left coronary artery ligation.^13^ MI or sham operations were performed randomly over the day. Prior to the surgery, mice (N = 66) were anaesthetised (i.p.; fentanyl 0.05 mg/kg; midazolam 5 mg/kg; medetomidine 0.5 mg/kg) and, before any incision was made, the adequacy of anaesthesia was monitored by testing rear foot reflexes. Respiratory pattern, rectal temperature, and responsiveness to manipulations were continually monitored throughout the procedure.

Seven days prior to termination (day 21 after the surgery), heart function was assessed with echocardiography in order to validate the presence and effect of MI in HF mice (Supplementary Fig. S2). HR, respiration and body temperature were constantly monitored, with body temperature kept between 36.0 and 38.0 °C using heating lamps. 2D images were recorded on the short axis of the heart on multiple levels in both end systole and end diastole, as well as with respiratory triggering. Subsequently, obtained images were used for complete 3D reconstruction of the heart. Image acquisition and analyses were performed using the dedicated Vevo® 2100 System and Software (Fujifilm VisualSonics Inc., Toronto, Canada).

On day 28, murine heart, kidneys, and liver were harvested at 7 AM = ZT1, 10 AM = ZT4, 1.30 PM = ZT7.5, 5 PM = ZT11, 8.30 PM = ZT14.5, midnight = ZT18, 3.30 AM = ZT21.5 from randomly chosen mice and snap frozen for quantitative real-time polymerase chain reaction (qPCR) analysis.

Since we sought to monitor biological processes in the mice and according to the 3R principle that required us to use as few animals as possible, we wanted to work with a homogenous mouse population (strain, age, sex, etc.) in order to lessen the biological differences between tested animals. We chose to work with C57BL/6 since we have a lot of experience using them for the left anterior descending artery (LAD) ligation model. Furthermore, they are a well-established mouse strain for the study of cardiovascular diseases and thus any findings will be relevant for those using the mice in cardiovascular preclinical studies. Males show less variation due to the absence of an estrous cycle^14^ and the majority of patients were also male, as is common in HF studies.

#### Gene expression analysis

Total RNA was isolated using 1 mL TripureTM Isolation Reagent (Roche) according to the manufacturer's protocol. Following DNAse I (Qiagen) treatment, 500 ng total RNA was used for cDNA synthesis (iScriptTM cDNA synthesis kit, Bio-Rad). qPCR reaction was performed using 10 μL iQTM SYBR Green supermix and 10 μL cDNA. Ribosomal Protein Lateral Stalk Subunit P0 (*Rplp0*) was selected as the housekeeping gene and was used for calculation of normalised gene expression levels (ΔCt). The sequences of all primers are presented in Supplementary Table S1.

### Study design: zebrafish heart failure

Wild-type AB zebrafish male adults aged 8–10 months were used in this study. Before proceeding with an experiment, fish from the same breeding stock were weighed and randomised into experimental tanks in a homogenous manner so that each tank contained the same number of fish with the same range of weight. Experimental tanks were maintained in a strict 14/10-h light/dark cycle environment (ZT0 = lights-on, ZT14 = lights-off) at 28 °C and animals were fed to satiety twice daily with dry food. After one week of acclimatisation, treatment with phenylhydrazine hydrochloride (PHZ, Sigma–Aldrich) was started to generate HF phenotype during five weeks. In brief, PHZ was added to the fish water of the treated group for 30 min every 3 days for five weeks. PHZ was used at a concentration of 1.25 μg/mL during the first week of treatment followed by a dose increment to 2.5 μg/mL for the rest of the protocol. Each treatment phase of 30 min incubation was followed by 30 min rinsing and washing in fish water without PHZ. The control group was subjected to the same process, excluding the addition of PHZ to the fish water. At the end of the five-week period, PHZ-treated and control fish were sacrificed at different time-points: 8:30 AM = ZT0, 12.30 PM = ZT4, 4.30 PM = ZT8, 8.30 PM = ZT12, 12.30 AM = ZT16, 4.30 AM = ZT20 (N = 108).

#### Gene expression analysis

Snap frozen organs were separately disrupted and homogenised by the Polytron Dispersing Aggregates (Kinematica) in the presence of QIAzol Lysis Reagent (Qiagen). Total RNA was isolated using the miRNeasy Micro Kit (Qiagen) according to the manufacturer's instructions. 500 and 1000 ng of DNase I treated (Qiagen) total RNA was reverse-transcribed using the Superscript II Reverse Transcriptase for heart and kidney samples, respectively (Invitrogen). qPCR reaction was carried out using the QuantiTect SYBR Green PCR Master Mix (Qiagen) and 10-fold dilutions of cDNA. Elongation factor 1-alpha 1 (*eef1a1*) was selected as the housekeeping gene and was used for calculation of normalised gene expression levels (ΔCt). The sequences of all primers are presented in Supplementary Table S1.

### Statistical analysis

For animal data, we performed a cosinor analysis, based on Cornelissen et al.^15^ by adjusting a macro provided by Doyle et al.^16^ to determine rhythmicity of delta Ct values. From the analyses we derived mesor, amplitude and acrophase values (with standard errors) and compared these between HF and control models. Note that acrophase of delta Ct value represents the trough of gene expression.

For the human data, we also performed a cosinor analysis, based on Cornelissen et al.^15^ by adjusting a macro provided by Doyle et al.^16^ We applied a log transformation for cTnT and melatonin because of skewed data, to achieve approximate normality. Cosinor analysis was fitted for every individual patient. Amplitude, acrophase and mesor were pooled within a multivariate (i.e. multiple outcomes) mixed model (similar to a meta-analysis approach, analysis designed as previously described by Sheu et al.1^17^). To obtain a normal distribution we have centred the acrophase for the troponin analysis around 09:00, which can be seen in all individual patients’ curves. Log-transformed values for mesor and amplitude were transformed back to a median with 95% confidence interval for interpretation, as previously described.^18^

**Fig. 1:**
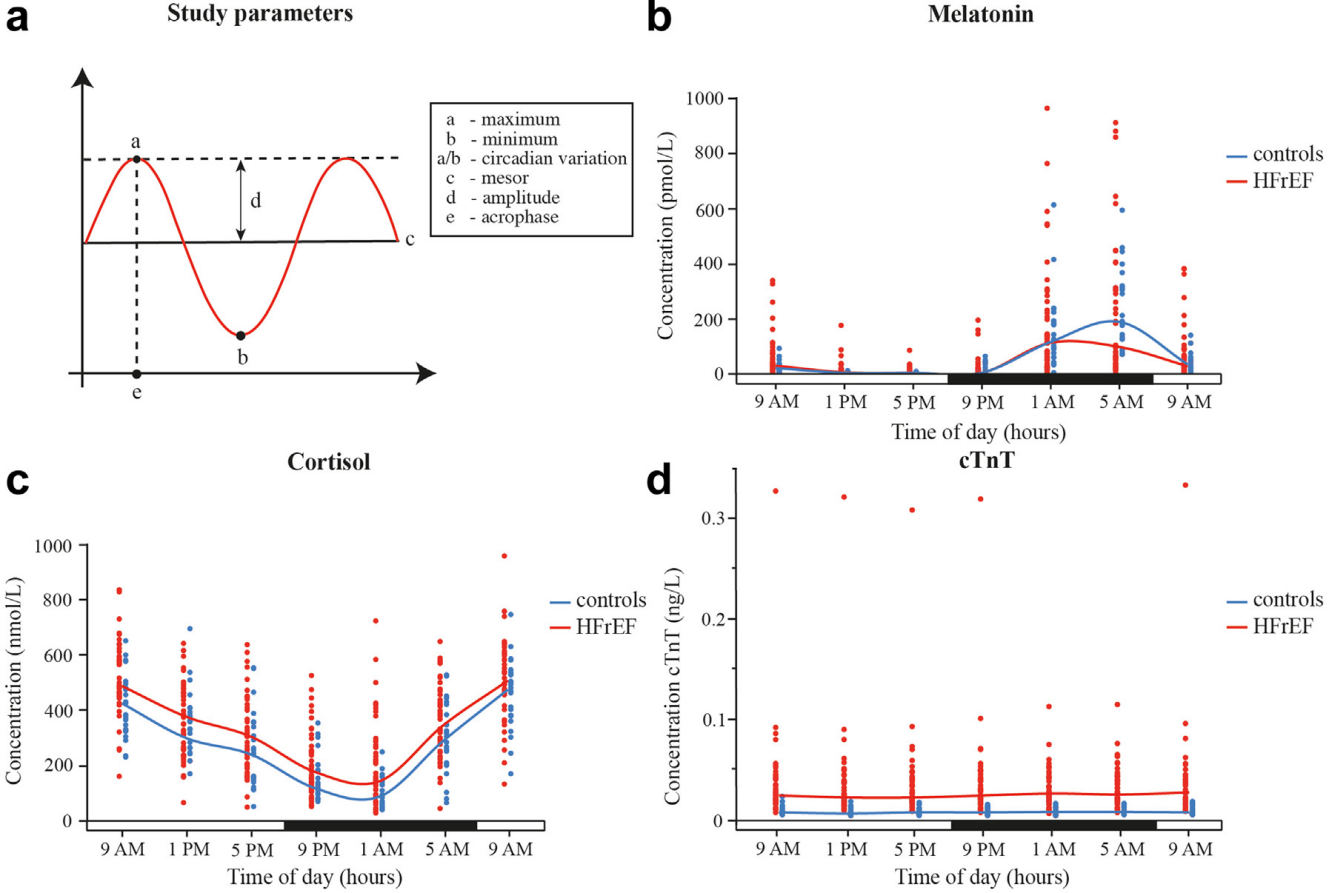
**24-hour hormonal output is intact in HFrEF patients.** (a) Parameters used for quantitative analysis of the 24-h melatonin and cortisol variation. 24-hour (b) melatonin (pmol/L), (c) cortisol (nmol/L) and (d) cTnT (ng/L) concentration. Each dot (red = patients, blue = controls) represents individual concentration at a given time-point. Lines indicate median values of all patients (red; N = 44–46) and controls (blue; N = 23–24). Horizontal bar indicates day (=white) and night (=black). cTnT = cardiac troponin T; HFrEF = heart failure with reduced ejection fraction.

Additionally, quantitative analyses were used to assess 24-h melatonin and cortisol variation: maximum, minimum, circadian variation (maximum/minimum; Fig. 1a). Melatonin values for single measurements below the detection limit (8 pmol/L) were assigned the value of 4 pmol/L. Three subjects with no detectable melatonin in all samples were excluded for the circadian analysis.

Linear regressions were performed (1) to assess an association between melatonin and cortisol levels and the presence of HF and (2) for associations for measurements in activity and sleep patterns. For minimum melatonin levels, many samples were undetectable (below 8 pmol/L), for which we performed a post-hoc dichotomization (detectable (n = 9) vs non-detectable (n = 61)) and subsequent logistic regression analysis with Firth's correction (to adjust for separation and to avoid small sample bias).

For all regular human analyses (cosinor and non-cosinor) correction for predefined confounders was performed (sex, age, body mass index (BMI), kidney function, and alcohol consumption), deliberately only including the most important variables as we are dealing with a small dataset.^19^ Smoking history was not included in our final analysis, as we encountered missing data on this variable. Sensitivity analyses were run with and without smoking data, which showed similar results in multivariable analyses.

Throughout the manuscript, the following indications for significance were used: ∗*P* < 0.05, ∗∗*P* < 0.01, ∗∗∗*P* < 0.001, ∗∗∗∗*P* < 0.0001. The human non-cosinor data was analysed in R studio v.2022.07.2 using the car^20^ and sjPlot^21^ packages. Cosinor analyses of the human and animal data were performed using SAS v.9.4 (SAS Institute Inc., Cary, NC, USA). Analysis scripts are available upon request.

The sample size of the mouse experiments was decided after performing a power calculation (power of 0.8, alpha of 0.05). From literature, on average 7 mice per time-point were used for the establishment of circadian rhythms in infarct size. From literature and previous experiments, we know that 30% variation in infarct size can be expected and that the LAD ligation has a 10% mortality rate, therefore increasing the total animals required for the study to 8 per group per time-point in studies comparing infarct size. For gene expression, we did a post hoc power calculation based on the delta Ct values and variation in HF animals. Based on a power of 0.8, alpha of 0.05 for a relevant difference in delta Ct of 20%, the minimum number of animals per group per time-point at the endpoint (sacrifice) was 3.

The heart and kidneys of three fish were pooled at each time-point and three samples were collected for both experimental conditions, totalling 9 PHZ-treated and 9 control fish per time-point. This number of pooled organs was previously found to allow sufficient concentrations (∼100 ng/μL) of good quality RNA (nucleic acid purity ratios 260/230 and 260/280 of ∼1.8–2.0) to be obtained for downstream qPCR analysis while considering the 3Rs rule.

### Role of funders

The funders did not have any role in study design, data collection, data analyses, interpretation, or writing of the report.

## Results

### Human heart failure: hormonal clock output is conserved in HF, with dampened oscillatory pattern

#### Study population characteristics

70 subjects were enrolled: 46 HFrEF patients (left ventricular ejection fraction (LVEF) <40% by echocardiography, NYHA II (32.6%) or III (67.4%)) and 24 age- and sex-matched controls (Table 1). The main reason for refraining from participation in this study was refusal to undergo additional tests. 71.7% of patients and 70.8% of controls were male, with a median age of 60 and 56 years, respectively. Renal function was similar between groups.

**Table 1:** Baseline characteristics.

	Patients (N = 46)	Controls (N = 24)	*P* value
Male (%)	71.7	70.8	1.0
Age, years	60 [14]	56 [16]	0.21
BMI, kg/m^2^	25.5 ± 4.6	24.9 ± 3.5	0.57
eGFR, mL/min/1.73 m^2^	70.2 ± 23.8	77.4 ± 21.7	0.22
**Medical history**			
Smoking, PY	2 [12.5]	0 [4.5]	0.041^b^
Alcohol, IU/week	0 [4]	7 [10]	0.056
Diabetes mellitus (%)	21.7	0.0	0.012^b^
Atrial fibrillation/flutter (%)	30.4	0.0	0.0015^b^
**Medication (%)**			
ACEi	58.7	16.7	0.001^b^
ARB	26.1	8.3	0.116
β-blocker	45.7	4.2	<0.001^b^
MRA	80.4	0.0	<0.001^b^
Amiodarone	26.1	0.0	0.006^b^
aBenzodiazepine	8.7	0.0	0.291
PPI	19.6	12.5	0.526
**Heart failure characterisation (%)**			
NYHA II	32.6	NA	NA
NYHA III	67.4	NA	NA
LVEF	22.7 ± 8.0	NA	NA
iCMP	43.5	NA	NA

Values are mean ± SD, median [interquartile range (IQR)] or percentage of total. Differences between the baseline characteristics of the patient and control group were compared by using the independent two-sample T-test for normal distributions and the Mann–Whitney U test for non-normal distributions (∗*P* < 0.05). ACEi = angiotensin converting enzyme inhibitor; ARB = angiotensin II receptor blocker; BMI = body mass index in kg/m^2^; eGFR = estimated glomerular filtration rate in mL/minute/1.73 m^2^ based on the 2021 CKD-EPI Creatinine equation; iCMP = ischemic cardiomyopathy; IU = international units; LVEF = left ventricular ejection fraction; MRA = mineralocorticoid receptor antagonist; NA = not applicable; NYHA = New York Heart Association functional classification; PPI = proton pump inhibitor; PY = packyears (one year of smoking 1 pack of cigarettes a day).

a Benzodiazepine medication was overall used by 8.7% of the patients, however not on the day of, and one day prior to, study interventions. Data was complete for all subjects, except for packyears which was missing in 1 case.

b Statistically significant difference: *P* < 0.05.

#### 24-h melatonin rhythmicity remains intact, but is dampened in heart failure

The rhythmic release of melatonin is under direct regulation of the central clock, thus reflecting any changes in its functionality. Patients and controls showed circadian variability of melatonin concentration, both in the pooled analysis (Fig. 1b) and on the individual level (Supplementary Fig. S3 and Table S2). Acrophases of 3:55 vs 03:41 (*P* = 0.43 [cosinor mixed model analysis]), and mesor of 21.9 vs 19.7 pmol/L (*P* = 0.63 [cosinor mixed model analysis]) did not differ between HFrEF patients and controls, while amplitude was significantly lower in HFrEF patients compared to controls of 5.2 vs 8.8 (*P* = 0.0001 [cosinor mixed model analysis]) (because of retransformation, these amplitudes have to be interpreted as multiplication factors in relation to mesor) (Table 2). In multivariable analyses, amplitude remained significantly lower in HFrEF patients compared to controls (*P* = 0.0002 [cosinor mixed model analysis]). For non-cosinor analyses, only 80.4% of patients reached a minimum value below the detection limit of <8 pmol/L while all controls did. Strikingly, circadian melatonin variation was significantly reduced in HF, indicating dampened output of the central circadian clock in the HF patients (Table 2).

**Table 2:** Melatonin, cortisol and cardiac troponin T in heart failure patients vs controls.

	Patients (N = 46)	Controls (N = 24)	Association with HF	Association with HF
	Mean ± SD/median [IQR]/median (95% CI)/%	Mean ± SD/median [IQR]/median (95% CI)/%	Unadjusted B (95% CI)	Adjusted B^a^ (95% CI)
**Melatonin** (pmol/L)				
Minimum	4.0 [0.0]	4.0 [0.0]	NA	NA
Maximum^b^	126.4 [261.5]	187.1 [261.5]	0.7 (0.4; 1.3)^c^	0.7 (0.4; 1.4)^c^
Circadian variation^b^	27.5 [40.5]	47.8 [48.6]	0.5 (0.3; 0.8)^c^^,^^f^	0.5 (0.4–0.8)^c^^,^^f^
Cosinor significance	93.2%	95.7%	NA	NA
Mesor^b^	21.9 (17.0; 28.1)^d^	19.7 (13.9; 28.0)^d^	1.1 (0.7; 2.1)^c^	1.1 (0.7; 1.7)^c^
Amplitude^b^, ^e^	5.2 (4.4; 6.0)^d^	8.8 (7.0; 10.9)^d^	0.6 (0.5; 0.8)^c^^,^^f^	0.6 (0.5; 0.8)^c^^,^^f^
Acrophase	03:55 (03:33; 04:17)	03:41 (03:12; 04:09)	00:14 (−00:22; 00:50)	00:06 (−00:28; 00:41)
**Cortisol** (nmol/L)				
Minimum^b^	132.4 [130.4]	77.1 [66.8]	1.6 (1.2; 2.1)^c^^,^^f^	1.4 (1.1; 1.9)^c^^,^^f^
Maximum	535.3 ± 119.7	501.1 ± 113.1	34.2 (−24.8; 93.3)	43.8 (−15.3; 102.9)
Circadian variation^b^	3.9 [2.9]	6.3 [3.9]	0.7 (0.5–0.9)^c^^,^^f^	0.7 (0.6–1.0)^c^^,^^f^
Cosinor significance	80.4%	87.5%	NA	NA
Mesor	331.9 (304.9; 359.0)	275.1 (237.3; 312.9)	56.8 (10.3; 103.3)^f^	56.0 (11.7; 100.3)^f^
Amplitude	165.5 (150.0; 181.0)	165.7 (143.7; 187.6)	−0.2 (−27.1; 26.7)	11.9 (−14.8; 38.7)
Acrophase	10:06 (09:41; 10:31)	09:55 (09:20; 10:31)	00:11 (−00:33; 00:54)	00:07 (−00:39; 00:52)
**cTnT**				
Minimum	0.0190 [0.0235]	0.0050 [0.0035]	NA	NA
Maximum	0.0260 [0.0260]	0.0070 [0.0064]	NA	NA
Circadian variation	1.3 [0.3]	NA	NA	NA
Cosinor significance	63%	NA	NA	NA
Mesor^b^	0.026 (0.021; 0.032)^d^	NA	NA	NA
Amplitude^b^, ^e^	1.107 (1.091; 1.124)^d^	NA	NA	NA
Acrophase	04:56 (03:27; 06:26)	NA	NA	NA

Melatonin, cortisol and cardiac troponin T levels shown as mean ± standard deviation (SD), median [interquartile range (IQR)], median (95% confidence interval (CI)). Minimum and maximum are the lowest and highest values determined during the 24-h blood sampling.

Circadian variation is calculated by dividing the maximum by the minimum. Effect of heart failure (HF) on minimum, maximum and circadian variation was assessed by multiple linear regression for melatonin and cortisol, except for minimum melatonin. Cosinor significance shows the percentage of subjects of which circadian variation of measured marker had a significant cosinor wave. Effect of heart failure on mesor, amplitude, and acrophase was assessed by cosinor mixed model analysis. Acrophase was defined by the time of day at which the fitted cosinor wave peaks. For all values N = 46 for patients and N = 24 for controls, except circadian variation of melatonin, for which N = 44 for patients and N = 23 for controls. B = Beta coefficient; NA = not applicable.

a Adjustments were performed for age, biological sex, body mass index, alcohol consumption, and kidney function.

b Since values were not normally distributed, ln transformation was performed before performing linear regressions/cosinor mixed model analysis.

c These values were retransformed, therefore the beta coefficient should be interpreted as a multiplication factor.

d These values were retransformed and should therefore be interpreted as medians (95%-CI).

e Because of retransformation the amplitude is to be interpreted as a multiplication factor in relation to the mesor.

f Statistically significant difference: *P* < 0.05.

After correction for potential confounders, circadian melatonin variation remained lower in patients than in controls (Table 2). Univariable logistic regression and multivariable linear regression analyses showed no significant association between HF and minimum and maximum melatonin levels. Interestingly, in the corrected analyses higher BMI was associated with lower maximum levels (*P* = 0.002 [multivariable linear regression]), lower circadian variation (*P* = 0.002 [multivariable linear regression]), lower mesor (*P* = 0.0154) and lower amplitude (*P* = 0.0084 [cosinor mixed model analysis]) of melatonin.

#### 24-h cortisol rhythmicity remains intact, but is relatively dampened, in heart failure

Cortisol represents another hormone which directly reflects the central clock output. Circadian variability was again confirmed for both groups (Fig. 1c, Supplementary Fig. S3 and Table S2). Cosinor analyses revealed similar acrophases of 10:06 vs 09:56 (*P* = 0.63 [cosinor mixed model analysis]) and amplitudes of 165.5 vs 165.7 nmol/L (*P* = 0.99 [cosinor mixed model analysis]) for HFrEF patients and controls, respectively (Table 2). Mesor was significantly elevated in HFrEF patients compared to controls, 331.9 vs 275.1 nmol/L (+56.8, *P* = 0.017 [cosinor mixed model analysis]), respectively (Table 2). In multivariable analyses, mesor remained significantly higher in HFrEF patients compared to controls while correcting for confounders (+56.0 nmol/L, *P* = 0.014 [cosinor mixed model analysis]) (Table 2). In non-cosinor analyses, minimum cortisol values were significantly higher in patients than in controls, while maximum values were similar in both groups. In line with an increased mesor, circadian cortisol variation was relatively reduced in HF patients, further confirming the dampened output of the central circadian clock in the HF patients (Table 2). After correction for confounders, circadian cortisol variation remained lower in patients than in controls (Table 2).

In the adjusted analyses, higher age was associated with higher minimum levels (*P* = 0.033 [multivariable linear regression]) and lower circadian cortisol variation (*P* = 0.043 [multivariable linear regression]). Higher alcohol consumption was associated with higher maximum levels (*P* = 0.043 [multivariable linear regression]), higher mesor (*P* = 0.0243 [cosinor mixed model analysis]) and higher amplitude (*P* = 0.0374 [cosinor mixed model analysis]) of cortisol.

#### HF patients have a high prevalence of non-dipping BP and HR

Both HR and BP are known to portray diurnally varying patterns, which are controlled by the circadian clock.^1^ The mean overall BP was 102/62 mmHg. The mean diurnal and nocturnal BP was 104/65 mmHg and 98/59 mmHg, respectively. Only eight patients (22.2%) had a dipper pattern (=at least 10% fall), while 21 (58.3%) had a non-dipper pattern and seven (19.4%) had a riser pattern.

A nocturnal HR dip was found in only eight (22.2%) patients, whereas 28 (77.8%) showed a non-dipping pattern.

#### Activity and sleep quality decline in HF

Since sleep–wake cycles and physical activity are interrelated with circadian rhythms, we sought to determine if changes in central clock output reflect on this aspect as well. Both peak and mean activity values were lower in patients, and patients had a significantly longer total sleeping time than controls, both in univariable analysis and multivariable analysis, correcting for potential confounders (mean difference 49 min, *P* = 0.005 [multivariable linear regression]). However, sleep interruption, latency and efficiency were not significantly different (Table 3).

**Table 3:** Activity and sleep characteristics of HFrEF patients and controls as measured by actigraphy.

	Patients (N = 36)	Controls (N = 23)	Effect of HF	Effect of HF
	Mean ± SD/median [IQR]	Mean ± SD/median [IQR]	Unadjusted B (95% CI)	Adjusted B^a^ (95% CI)
Peak activity^b^ (counts per minute)	2027.0 [1106.5]	2413.0 [1043.0]	0.8 (0.6; 0.9)^e^	0.8 (0.6; 0.9)^e^
Mean activity (counts per minute)	129.8 ± 45.8	220.9 ± 57.5	−91.1 (−118.1; −64.0)^e^	−87.5 (−116.0; −59.0)^e^
	*N* = *34*	*N* = *22*		
Total sleep time (minutes)	443.7 ± 61.9	394.9 ± 58.4	48.8 (15.6; 82.0)^e^	48.2 (13.5; 82.9)^e^
Sleep latency^b^, ^c^ (minutes)	9.5 [17.5]	7.0 [14.3]	1.1 (0.6; 1.9)	1.1 (0.6; 1.9)
WASO^b^ (minutes)	58.0 [27.0]	46.0 [26.5]	1.2 (0.9; 1.5)	1.2 (0.9; 1.5)
Sleep efficiency^d^ (%)	84.0 [7.3]	83.5 [9.3]	15.7 (−21.1; 30.6)	13.8 (−23.2; 30.4)

Values are mean ± SD or median [interquartile range (IQR)]. Beta coefficients (B) are presented with their 95% confidence interval (CI). The effect of heart failure on actigraphy parameters was assessed using linear regression. HF = Heart failure; WASO = Wake after sleep onset.

a Adjustments were performed for age, biological sex, body mass index, alcohol consumption, and kidney function.

b Since values were not normally distributed, natural logarithm transformation was performed for the regression analyses. Beta values shown were retransformed and should be interpreted as multiplication factors.

c Since values of 0 were present, the value of 1 was added to all data points before performing logistic transformation.

d Quadratic transformation was applied in order to achieve approximate normality before performing regression analyses. Beta coefficients were retransformed.

e Statistically significant difference: *P* < 0.05.

Furthermore, 11.4% of patients experienced excessive daytime sleepiness, compared to none of the controls. No differences were identified regarding chronotype distribution in both groups.

Interestingly, patients reported a strongly reduced subjective sleep quality compared to controls: patients more often experienced difficulty falling asleep, felt that they woke up more frequently during the night, and more often reported not to feel rested in the mornings (Table 4). These discrepancies remained present in a sensitivity analysis of only the 54 subjects for whom both questionnaires and actigraphy were available, although the difference in waking up during the night became less pronounced (*P* = 0.057 [Fisher's exact test, two-sided]; data not shown).

**Table 4:** Activity and sleep questionnaire results of HFrEF patients and controls.

	Patients (N = 46)	Controls (N = 24)	*P* value
**ESS (%)**			
	*N* = *44*	*N* = *24*	
Normal daytime sleepiness	88.6	100.0	0.15
Excessive daytime sleepiness	11.4	0.0	
**Difficulty falling asleep (%)**			
Never to sometimes	63.6	91.7	0.020^a^
Often to very often	36.4	8.3	
**Waking up during the night (%)**			
Never to sometimes	38.6	70.8	0.021^a^
Often to very often	61.4	29.2	
**Not rested in the mornings (%)**			
Never to sometimes	56.8	100.0	<0.0001^a^
Often to very often	43.2	0.0	
**VOA questionnaire (%)**			
	*N* = *43*	*N* = *24*	
Morning chronotype	46.5	45.8	0.58
Evening chronotype	2.3	8.3	
None of both	51.2	45.8	

Fisher's exact test was utilised to determine the existence of non-random associations between the dichotomous values of patients compared with controls.

ESS = Epworth Sleepiness Scale; VOA-questionnaire = Vragenlijst Ochtend/Avond typering (translation: questionnaire morning/evening chronotype).

a Statistically significant difference: *P* < 0.05.

#### cTnT exhibits a circadian rhythm in HF patients

To obtain information on heart-specific circadian rhythmicity, 24-h serum cTnT concentrations were analysed. In HF patients, cosinor cTnT analysis revealed an acrophase of 4:56, a mesor of 0.026 ng/L and an amplitude of 1.1 (because of retransformation, amplitude has to be interpreted as a multiplication factor in relation to mesor) (Table 2, Fig. 1d). In line with previous studies reporting circadian oscillations of cTnT concentration in healthy subjects,^11^ we visually observed a similar oscillatory pattern in our controls although not statistically confirmed due to the insufficient sensitivity of the assay when measuring very low analyte concentrations (data not shown).

cTnT concentrations exhibited a normal circadian rhythm in HF patients (Supplementary Fig. S3), suggesting preservation of the molecular clock in the heart. However, since no unequivocal cellular rhythm can be detected directly in human cardiac tissue without repeated tissue sampling, we next used animal HF models to further confirm the *in-situ* preservation of circadian rhythms in the failing heart.

### The peripheral circadian clock is conserved in heart failure in mice

Chronic HF was confirmed by gene expression analysis of natriuretic peptide type A (*Anp*) and brain natriuretic peptide (*Bnp*) in murine heart tissue (Supplementary Fig. S4).

**Fig. 2:**
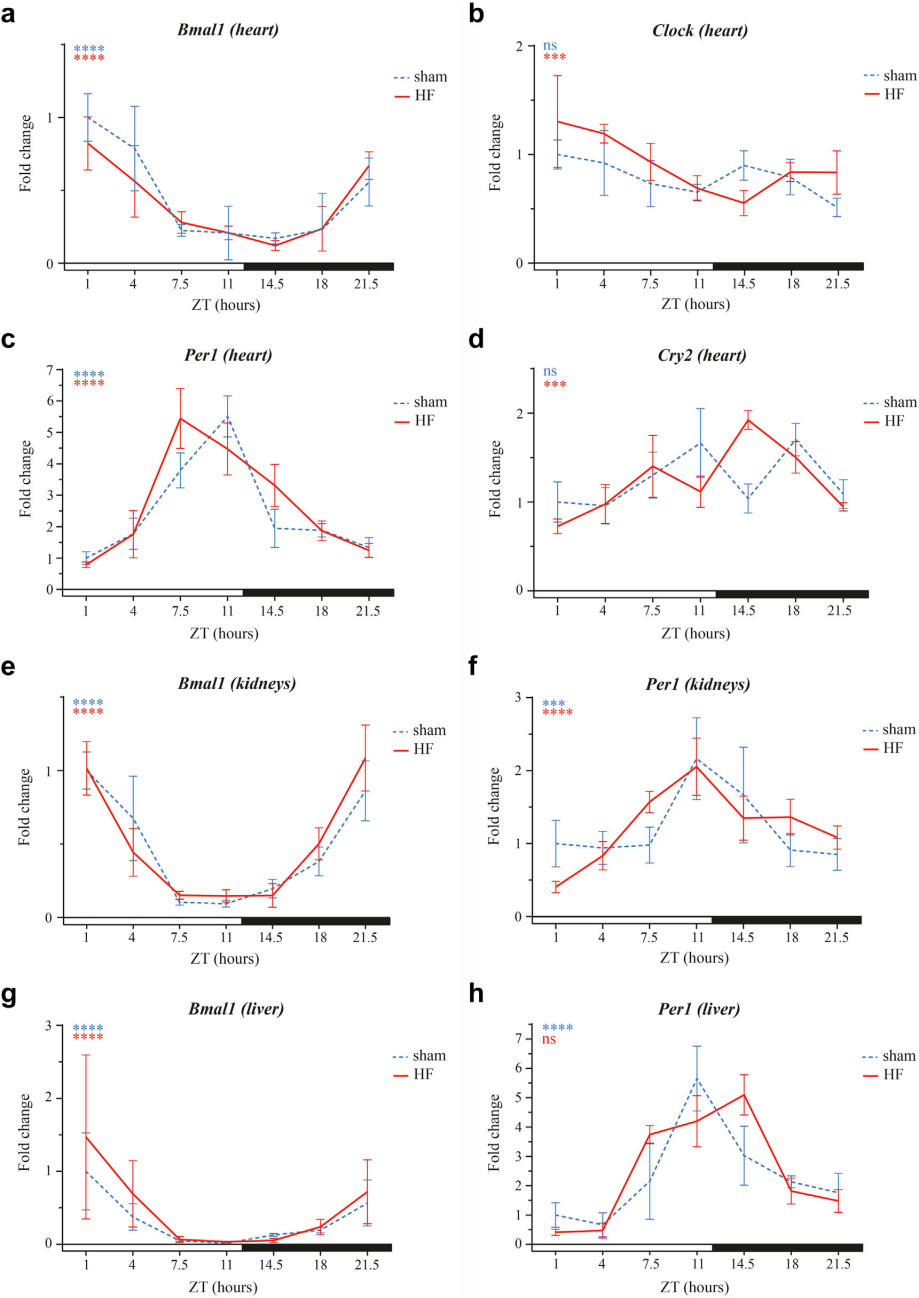
**24-hour expression of core clock genes in murine organs.** 24-hour (a) *Bmal1*, (b) *Clock*, (c) *Per1*, and (d) *Cry2* expression levels in murine heart, and (e, g) *Bmal1* and (f, h) *Per1* in (e, f) kidneys and (g, h) liver between sham and HF group (N = 3–5 mice/group/ZT; mean ± SEM), normalised against sham ZT1. Statistical significance of cosinor rhythmicity of delta Ct values per group is indicated at the top left graph corner: ∗∗∗*P* < 0.001, ∗∗∗∗*P* < 0.0001. Horizontal bar indicates lights-on (=white; ZT0) and lights-off (=black; ZT12) period. *Bmal1* = Brain and Muscle ARNT-Like 1; *Clock* = Circadian Locomotor Output Cycles Kaput; *Cry2* = Cryptochrome Circadian Regulator 2; HF = heart failure; *Per1* = Period Circadian Regulator 1; ZT = zeitgeber time.

Core clock genes *Bmal1* and *Per1*, displayed statistically significant circadian cosinor-shaped oscillations in both sham and HF murine hearts (*P* < 0.0001 in each group; Fig. 2a–c [cosinor analysis]). The same was true for almost all other tested genes, except for cardiac *Clock* and *Cry2* expression in sham models and *Per1* expression in the liver of HF models (Fig. 2a–h). Cosinor analysis revealed similar cosinor parameters (mesor, amplitude, acrophase) between HF and sham models for almost all clock-related genes in the heart, kidneys and liver. However, the mean amplitude of *Clock* expression in the heart was slightly lower (delta Ct value 0.4 higher) in HF than in sham models (95% confidence interval of delta Ct value: 0.1; 0.8). Also, the phase of *Per1* delta Ct values in the liver was 03:55 h shifted in HF compared to sham models (95%-confidence interval of delta Ct value: 02:17; 10:07) (Table 5).

**Table 5:** Cosinor analysis of mouse gene expression.

	HF (95%-CI)	Sham (95%-CI)	B-coefficient (95%-CI)
**Heart**			
*Per1*			
Mesor	3.9 (3.7; 4.2)	4.0 (3.8; 4.3)	−0.1 (−0.4; 0.2)
Amplitude	1.3 (0.9; 1.6)	1.0 (0.7; 1.4)	−0.2 (−0.2; 0.7)
Acrophase	01:32 (00:36; 02:27)	01:36 (00:27; 02:45)	−00:04 (−00:56 00:46)
Cosinor wave	S	S	NA
*Bmal1*			
Mesor	9.6 (9.3; 9.9)	9.5 (9.2; 9.9)	0.1 (−0.4; 0.6)
Amplitude	1.3 (0.8; 1.7)	1.3 (0.8; 1.8)	−0.0 (−0.7; 0.6)
Acrophase	22:53 (21:07; 00:39)	22:59 (21:34; 00:25)	−00:06 (−02:31; 02:19)
Cosinor wave	S	S	NA
*Clock*			
Mesor	6.8 (6.6; 7.0)	7.0 (6.8; 7.2)	−0.2 (−0.4; 0.1)
Amplitude	0.5 (0.3; 0.8)	0.1 (−0.2; 0.4)	0.4 (0.1; 0.8)^a^
Acrophase	03:59 (23:56; 08:01)	21:33 (08:43; 10:23)^b^	06:25 (−07:26; 20:17)
Cosinor wave	S	NS	NA
*Cry2*			
Mesor	4.9 (4.7; 5.0)	4.8 (4.6; 5.0)	0.1 (−0.2; 0.3)
Amplitude	0.5 (0.2; 0.7)	0.3 (−0.0; 0.5)	0.2 (−0.1; 0.6)
Acrophase	19:58 (12:55; 03:01)	22:19 (18:23; 02:14)	−02:20 (−11:04; 06:23)
Cosinor wave	S	NS	NA
**Kidney**			
*Per1*			
Mesor	4.0 (3.7; 4.2)	3.9 (3.7; 4.2)	0.0 (−0.3; 0.4)
Amplitude	0.8 (0.5; 1.2)	0.6 (0.2; 0.9)	0.3 (−0.2; 0.7)
Acrophase	23:13 (19:55; 02:31)	00:02 (21:44; 02:21)	−00:49 (−05:09; 03:30)
Cosinor wave	S	S	NA
*Bmal1*			
Mesor	6.0 (5.8; 6.3)	6.2 (5.9; 6.4)	−0.2 (−0.5; 0.2)
Amplitude	1.6 (1.3; 2.0)	1.8 (1.4; 2.2)	−0.2 (−0.7; 0.4)
Acrophase	01:00 (00:25; 01:36)	00:58 (00:11; 01:44)	00:02 (−00:42; 00:48)
Cosinor wave	S	S	NA
**Liver**			
*Per1*			
Mesor	12.6 (0.9; 24.3)	4.6 (−7.3; 16.4)	8.0 (−8.6; 24.6)
Amplitude	14.7 (−1.7; 31.1)	1.2 (−15.4; 17.7)	13.5 (−9.7; 36.8)
Acrophase	02:43 (01:20; 06:47)	22:48 (14:54; 06:43)^b^	03:55 (02:17; 10:07)^a^
Cosinor wave	NS	S	NA
*Bmal1*			
Mesor	7.7 (7.3; 8.1)	8.0 (7.6; 8.4)	−0.3 (−0.9; 0.3)
Amplitude	2.8 (2.1; 3.4)	2.5 (1.9; 3.1)	0.3 (−0.6; 1.2)
Acrophase	00:08 (23:02; 01:14)	00:51 (23:57; 01:45)	−00:43 (−02:06; 00:39)
Cosinor wave	S	S	NA

Cosinor parameters for dCt values of mice with heart failure (HF) and sham mice, with their respective 95% confidence intervals (95%-CI). The B-coefficients represent the difference between the animal models (HF—sham) and the 95%-CI represents the level of statistical significance of the difference. The acrophase represents clock time in the HF and control columns, while the B-coefficient column represents the time difference. Note that acrophase of delta Ct value represents the trough of gene expression.

NA = Not applicable, NS = Non-significant cosinor wave (*P* ≥ 0.05), S = Significant cosinor wave (*P* < 0.05).

a Statistically significant difference: *P* < 0.05.

b Width of confidence interval exceeds 24 h.

Taken together, both groups had the same expected pattern of clock gene oscillation in all three organs: *Bmal1* and *Clock* oscillated in the same phase, with the highest expression during the beginning of the rest phase (=day), gradually declining towards the active phase, while *Per1* and *Cry2* had the opposite peak and trough time (‘anti-phase’).

Although the mouse model of HF is well-established, mouse is a nocturnal animal with rest/activity cycles diametrically opposite from diurnal humans. Therefore, we also used diurnal zebrafish to confirm our findings.

### The peripheral circadian clock is conserved in heart failure in zebrafish

Chronic HF was confirmed by gene expression analysis of *anp* and *bnp* in zebrafish heart tissue (Supplementary Fig. S5).

**Fig. 3:**
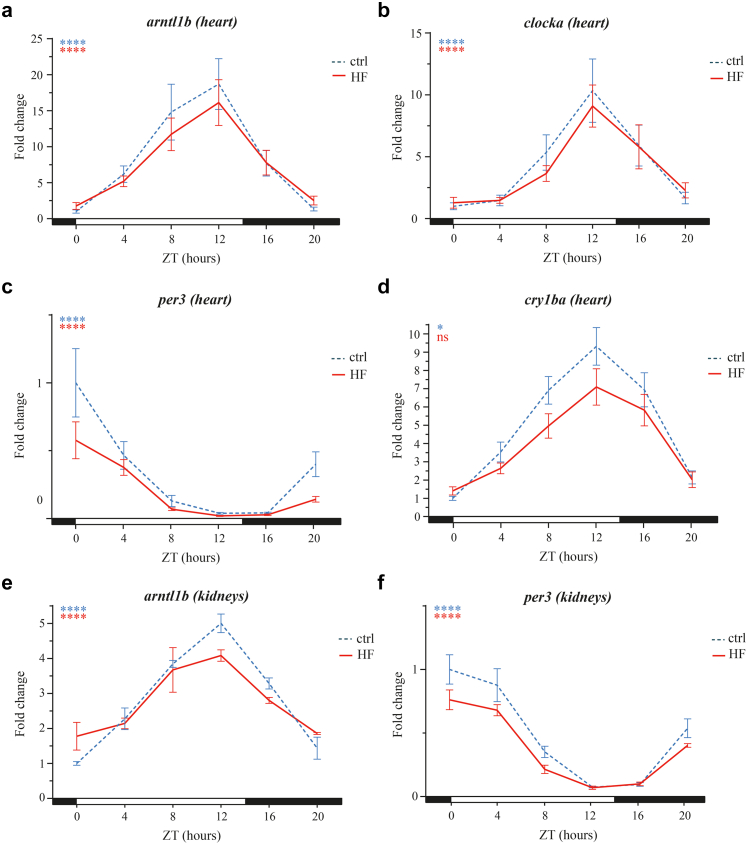
**24-hour expression profiles of core clock genes in zebrafish organs.** 24-hour (a) *arntl1b*, (b) *clocka*, (c) *per3*, and (d) *cry1ba* expression levels in zebrafish heart, and (e) *arntl1b* and (f) *per3* in kidneys between control and HF ((a–d) N = 9, (e) N = 3, (f) N = 6 zebrafish/group/ZT; mean ± SEM), normalised against control ZT0. Statistical significance of cosinor rhythmicity of delta Ct values per group is indicated at the top left graph corner: ∗*P* < 0.05, ∗∗∗∗*P* < 0.0001. Horizontal bar indicates lights-on (=white; ZT0) and lights-off (=black; ZT14) period. *arntl1b* = Aryl Hydrocarbon Receptor Nuclear Translocator Like 1 b. Other abbreviations as in Fig. 2.

Core clock genes, *arntl1b* (=*Bmal1*), *clocka*, and *per3*, displayed significant oscillations in both control and HF zebrafish hearts (all *P*-values <0.0001; Fig. 3a–c [cosinor analysis]). *Cry1ba* only had a statistically significant cosinor pattern in control zebrafish, but not in HF models (*P* = 0.027 vs *P* = 0.134; Fig. 3d [cosinor analysis])*.*

In kidneys, *arntl1b* and *per3* showed cosinor oscillations in both HF and control zebrafish (all *P-*values <0.0001; Fig. 3e and f [cosinor analysis]). Table 6 shows similar cosinor parameters for HF and control zebrafish. For cardiac *per3* expression, mean expression was slightly lower (mesor delta Ct value 0.8 higher) in HF zebrafish than controls (95% confidence interval of delta Ct value: 0.3; 1.4) and for *arntl1b* in kidneys, the phase of delta Ct values was shifted by 01:04 h in HF compared to control zebrafish (95% confidence interval of delta Ct value: 00:06; 02:01).

**Table 6:** Cosinor analysis of zebrafish gene expression.

Heart	HF (95%-CI)	Control (95%-CI)	B-coefficient (95%-CI)
*per3*			
Mesor	10.1 (9.7; 10.4)	9.2 (8.8; 9.6)	0.8 (0.3; 1.4)^a^
Amplitude	2.5 (2.0; 3.0)	2.5 (1.9; 3.0)	0.1 (−0.7; 0.8)
Acrophase	20:08 (18:52; 21:24)	19:35 (18:55; 20:15)	00:33 (−00:43; 01:50)
Cosinor wave	S	S	NA
*arntl1b*			
Mesor	9.7 (9.3; 10.1)	9.8 (9.4; 10.2)	−0.2 (−0.7; 0.4)
Amplitude	1.6 (1.1; 2.1)	2.2 (1.7; 2.8)	−0.6 (−1.4; 0.1)
Acrophase	23:00 (21:48; 00:12)	23:05 (22:09; 00:01)	−00:04 (−01:42; 01:32)
Cosinor wave	S	S	NA
*clocka*			
Mesor	10.4 (9.9; 10.8)	10.4 (9.9; 10.8)	−0.0 (−0.7; 0.7)
Amplitude	1.4 (0.8; 2.1)	1.7 (1.1; 2.4)	−0.3 (−1.2; 0.6)
Acrophase	21:13 (18:51; 23:35)	21:17 (19:54; 22:41)	−00:04 (−02:51; 02:43)
Cosinor wave	S	S	NA
*cry1ba*			
Mesor	7.7 (6.6; 8.8)	8.0 (6.9; 9.1)	−0.3 (−1.8; 1.2)
Amplitude	1.7 (0.2; 3.3)	1.5 (−0.1; 3.0)	0.3 (−0.5; 1.2)
Acrophase	01:45 (00:0; 03:22)	00:28 (20:28; 04:28)	01:16 (−01:56; 04:30)
Cosinor wave	NS	S	NA
**Kidneys**			
*per3*			
Mesor	9.2 (8.9; 9.4)	8.8 (8.6; 9.1)	0.3 (−0.0; 0.7)
Amplitude	1.8 (1.5; 2.2)	2.0 (1.7; 2.4)	−0.2 (−0.7; 0.3)
Acrophase	20:15 (18:49; 21:41)	00:28 (20:28; 04:28)	−00:24 (−02:01; 01:11)
Cosinor wave	S	S	NA
*arntl1b*			
Mesor	9.4 (9.3; 9.5)	9.4 (9.3; 9.6)	−0.0 (−0.2; 0.1)
Amplitude	0.7 (0.5; 0.8)	1.1 (0.9; 1.3)	−0.4 (−0.6; −0.2)
Acrophase	23:13 (22:33; 23:54)	22:09 (21:31; 22:48)	01:04 (00:06; 02:01)^a^
Cosinor wave	S	S	NA

Cosinor parameters for dCt values of zebrafish with heart failure (HF) and control zebrafish, with their respective 95% confidence intervals (95%- CI). The B-coefficients represent the difference between the animal models (HF—control) and the 95%-CI represents the level of statistical significance of the difference. The acrophase represents clock time in the HF and control columns, while the B-coefficient column represents the time difference. Note that acrophase of delta Ct value represents the trough of gene expression.

NA = Not applicable, NS = Non-significant cosinor wave (*P* ≥ 0.05), S = Significant cosinor wave (*P* < 0.05).

a Statistically significant difference: *P* < 0.05.

As observed in mice, both zebrafish groups had the same expected pattern of clock gene oscillation: *arntl1b* and *clocka* oscillated in the same phase, while *per3* was in ‘anti-phase’*. Cry1ba*, one of many variants of the zebrafish *cry* gene, oscillated in the same phase as *arntl1b* and *clocka*, as previously shown.^22^^,^^23^ *Arntl1b*, *clocka*, and *cry1ba* expression gradually increased during the day and peaked at the end of the active phase (=day) and beginning of the rest phase (=night), while *per3* expression was opposite. Furthermore, being diurnal animals, oscillations in zebrafish were expectedly in opposite phases to those of nocturnal mice.

## Discussion

Here, we show that circadian rhythms remain present in HF, although with less robust central hormonal output in human patients as reflected by dampening of the typical oscillatory patterns of melatonin and cortisol. We found no influence of HF on the presence of a cosinor pattern of these hormones in individual patients. HF patients had relatively lower nighttime melatonin peaks than controls, while their cortisol levels remained chronically elevated throughout the day and night. Congruently, the normal nighttime dip in BP and HR was present in only a small minority of patients. HF patients were less physically active and reported reduced subjective sleep quality, although objective sleep parameters were similar and they even slept longer than controls.

Although there is no comparable data available on multi-species mapping of circadian rhythmicity in HF, dampening in the 24-h concentrations of clock-related hormones was previously demonstrated in end-stage renal disease.^8^ Furthermore, dampened circadian rhythms and suprachiasmatic nucleus dysfunction have been linked with ageing.^24^

The high prevalence of non-dipping BP and HR patterns in our HF population is in line with previous studies.^25^^,^^26^ In hypertensive non-dippers this has been correlated to impaired nocturnal melatonin production^27^ and lower diurnal cortisol variation.^28^ Potential mechanisms through which melatonin influences BP and HR include its sympatholytic activity, nephroprotective action, and peripheral vasodilating properties.^29^ For cortisol, increased levels lead to an activation of the mineralocorticoid receptor, which in turn leads to sodium and water retention, and consequently, a rise in blood pressure besides sympathetic activation.^30^ As also discussed in a recent review by Kario and Williams, future studies may be directed to investigate whether modulation of 24-h rhythmicity in HF patients improves prognosis.^31^ While cortisol is –indirectly– targeted in current HF guideline-recommended therapy (e.g. beta-blockers, MRA), the potential of timed suppression to restore its 24-h circadian variation has not been investigated. Melatonin is not used in the current therapeutic setting, although supplementation has been shown to lower nocturnal BP.^32^^,^^33^

Interestingly, more alcohol consumption was associated with a higher maximum cortisol value. Previous studies have found that alcohol consumption is linked to circadian rhythm disruption and specifically, can cause plasma cortisol levels to increase with acute consumption, dependence, and withdrawal.^34^ However, other studies were not able to confirm this association.^34^ For melatonin, we found that a higher BMI was associated with lower maximum values and lower diurnal variation, which is in line with previous research pointing towards anti-obesogenic effects of melatonin, with possible mechanisms being energy store regulation, sleep–wake cycle control and influence on composition of gut microbes.^35^

Poor subjective sleep quality and lower physical activity in HF patients opens another possibility to improve function of the central clock through behavioural interventions. As observed in treating depression with light therapy and exercise,^36^^,^^37^ utilising this type of intervention could improve quality of life or even objective clinical outcomes for HF patients as well. In HF patients, insomnia has a prevalence of ∼33%.^38^ Although our actometer data did not show decreased sleep efficiency, HF patients did report lower subjective sleep quality. Poor sleep experience might lead to higher sympathetic nervous system activity and stress levels, negatively affecting disease progression. Insomnia and non-restorative sleep have been correlated with increased risk of incident HF.^4^ Our findings point to a need to address subjective sleep experience rather than actual sleep duration or interruption in HF patients.

Lastly, we found no differences between chronotype distribution in our groups. These findings, together with our findings on intact serum melatonin and cortisol circadian patterns, confirm the absence of phase shifting in the circadian rhythmicity of HF patients.

In humans, central clock output can be studied via 24-h fluctuations of melatonin and cortisol. However, a peripheral clock, specific for each individual tissue in the body (e.g. heart), is frequently inaccessible for repeated sampling. Therefore, research of human organs is usually performed at only one time-point per subject, either during a surgical procedure, or from deceased donors.^39^ This results in clustering of samples from different origin and time period, that mimic a 24-h pattern of organ-specific clock genes expression. Although informative, it does not provide important information on intraindividual circadian fluctuation.

Therefore, we substituted biopsies with a representative cardiac biomarker, measurable from serum: cTnT. Previous studies have shown that cTnT exhibits circadian variation in both healthy and diabetic subjects.^10^^,^^11^^,^^40^ Similarly, we observed significant 24-h oscillations in HF patients, pointing towards a preserved circadian rhythm in the failing heart. Furthermore, we have recently shown that soluble ST2 biomarker,^41^ reflecting myocardial wall stress and activation of the fibrosis pathway, also exhibits 24-h fluctuation in heart failure patients. This is in line with our current finding of a functional peripheral clock in heart failure.

Intact peripheral clocks are also confirmed in our animal HF models. In order to circumvent the limitations of repeated human organ sampling, we further corroborated our findings in the murine and zebrafish tissues, and found nearly complete overlap between HF and control groups, with only minor differences in single clock gene expression levels or acrophase. This is consistent with our observation of intact cTnT oscillation in human patients and in line with a previously published study conducted on orthotopic heart transplantation samples, where no difference in core clock gene expression was found between healthy controls, patients with coronary heart disease, and patients with cardiomyopathy.^42^ In addition, McTiernan et al.^43^ found intact gene expression of core clock genes as well as several ion channel genes in ventricles from failing human hearts, further corroborating our findings. Instead of repeated sampling within one subject, as we did, the previous studies pooled one time-point per each explanted end-stage failing heart, thus creating a signature of a time period. Although these approaches are intrinsically hampered by interindividual variation, the results corroborate our findings. Altogether, our results demonstrate the preservation of the molecular circadian clock in the heart, and in two other organs affected by HF (liver and kidney). While our current study specifically addresses chronic HF, we previously found intact clock gene oscillations in acute myocardial injury as well, indicating that the peripheral clock likely stays intact over the full period between the acute and chronic phases of HF.^44^

Although seemingly contra-intuitive at first, previous experiments using ablation of the SCN or its mediators have shown that indeed peripheral molecular clocks can function and stay synchronised even in the absence of central regulation, for example via food or exercise as Zeitgebers.^45^^,^^46^ Congruently, it seems plausible that dampened central circadian signalling may suffice for entrainment of peripheral circadian rhythms, compatible with a high evolutionary priority.

### Study limitations

We did not use standardised regimes commonly utilised in clinical circadian experiments regarding participants’ physical activity, food intake, light exposure, and sleep duration, to minimise external influences on circadian clock and to achieve a more comparable circadian rhythm between subjects.^10^ We deemed it necessary to allow for a clinically more translatable setup, since patients outside the study framework will not follow these externally imposed regimes and strictly controlled environmental settings. Consequently, all consistent differences found in central circadian clock output between our study subjects are more robust and applicable to real-life situations.

Other limitations include the low sample size compared to large clinical intervention trials. However, this study offers unique 24-h measurements during seven time-points per each subject, which has not been reported previously in HF. This, in our opinion, is a valuable addition, especially since most clinical HF studies disregarded circadian rhythms altogether or were performed by pooling only one time-point of each study participant with a categorical division into two or four broad time windows.^42^ Our subjects were predominantly white males and we used male animals, which is in line with other studies in HFrEF, but may reduce the generalisability to non-white and female patients, although in our multivariable analyses biological sex was not associated with any of the studied outcome measures.

Lastly, no hormonal output was measured in our animal models for practical reasons. Since the main goal of this study was to investigate the relationship between HF and circadian rhythms in humans, we used animals only to study the questions that cannot be answered in human material (organ tissue) and focussed all our efforts in preserving the quality of the collected tissue.

### Conclusions

Peripheral clock machinery is preserved in HF, with expression comparable to that of healthy controls. However, the central circadian clock output of HF patients is altered, resulting in a dampened oscillatory pattern of melatonin and cortisol. Furthermore, the prevalence of non-dipping BP and HR in HF patients is high and associated with lower nocturnal melatonin levels and higher nocturnal cortisol levels.

The fact that a significant amount of clock-governed variation remains intact in severely ill patients, here with HF, underlines the physiological importance of this regulatory system, and implies that the timing of interventions and diagnostic measurements should be taken into account in preclinical and clinical research. Finally, discovery of the aberrant function of specific clock components opens an exciting window of opportunity to develop new interventions and strategies to tackle HF (Central Illustration).

**Central Illustration:**
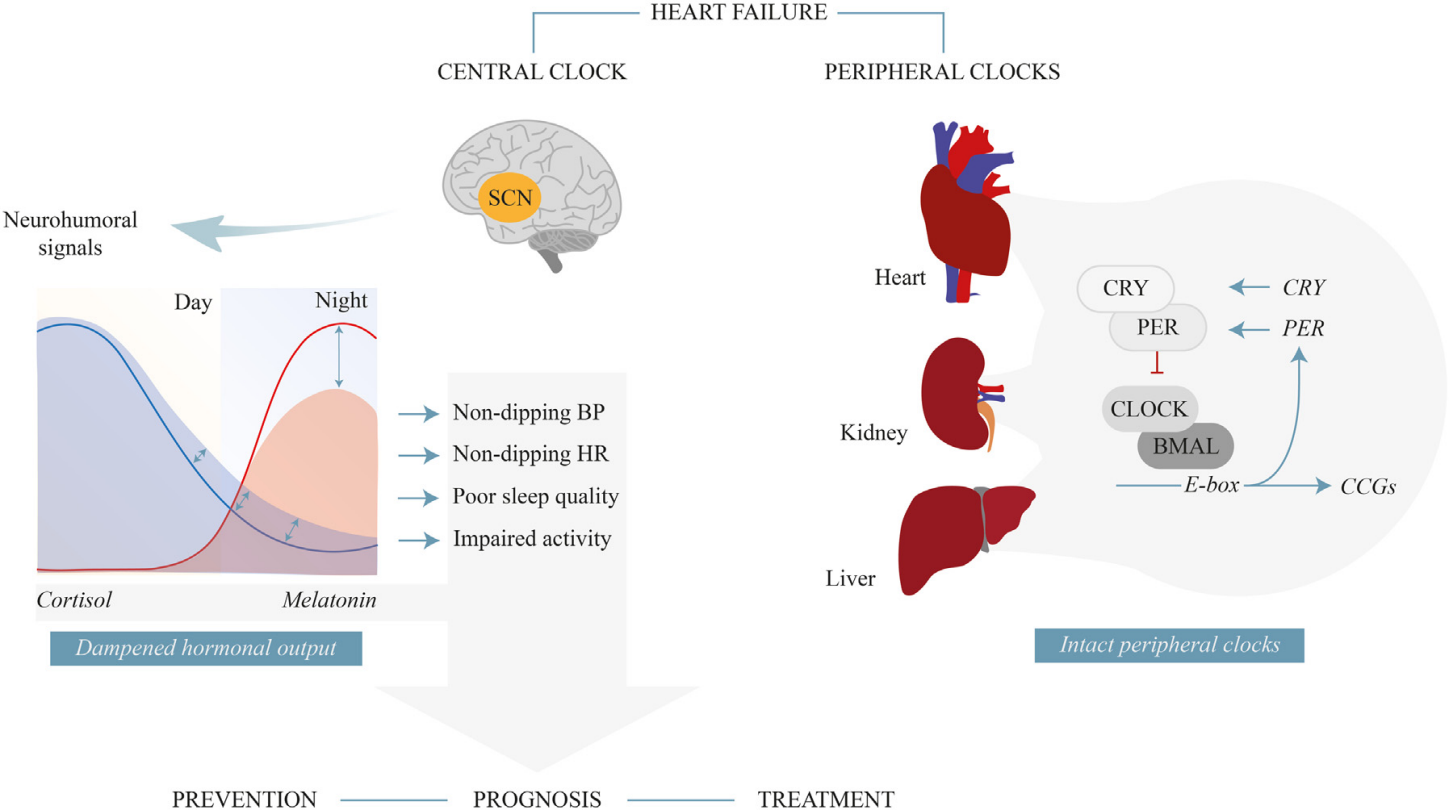
In HF, molecular clocks of heart, kidney and liver are preserved, with expression comparable to that of healthy controls. Conversely, HF negatively affects the central clock output: 24-h concentrations of melatonin and cortisol are dampened in HF, when compared to healthy controls. The prevalence of non-dipping BP and HR patterns in the HF population is high, correlating with lower nocturnal melatonin levels and higher nocturnal cortisol levels. HF patients suffer from poor subjective sleep quality and impaired activity. Altogether, these findings open various opportunities for developing new therapeutic (clock) interventions in order to improve the long-term quality of life and survival of patients suffering from HF, and warrant further investigations (e.g. melatonin supplementation, prognosis/prevention strategies based on non-dipping BP and HR, behavioural interventions for restoring subjective sleep quality and physical activity: light and exercise therapy, chronopharmacology). BMAL, Brain and Muscle ARNT-Like; BP, blood pressure; CLOCK, Circadian Locomotor Output Cycles Kaput; CRY, Cryptochrome Circadian Regulator; HF, heart failure; HR, heart rate; PER, Period Circadian Regulator; SCN, suprachiasmatic nucleus

## Contributors

L. W. van Laake, Y. Devaux conceived and designed the study; S. Crnko, M. I. Printezi, L. Leiteris, A. I. Lumley, L. Zhang, I. Ernens, T. P. J. Jansen, L. Homsma, D. Feyen, M. van Faassen, B. C. du Pré, and H. Kemperman collected the data; S. Crnko, M. I. Printezi, P. P. M. Zwetsloot, N. P. A. Zuithoff, and A. M. May performed data analysis; S. Crnko and M. I. Printezi wrote the manuscript; C. A. J. M. Gaillard, M. I. F. J. Oerlemans, P. A. F. M. Doevendans, A. M. May, J. P. G. Sluijter, Y. Devaux, and L. W. van Laake critically revised the manuscript. L. W. van Laake and Y. Devaux verified the underlying data of the manuscript. All authors provided approval of the final version for submission, and take responsibility for the accuracy and integrity of the data.

## Data sharing statement

Upon reasonable request, deidentified participant data, experimental data, and data dictionary can be shared with other researchers. Data will be made available after approval of a study proposal and after signing a data access agreement. Data sharing will be possible until 36 months after article publication. Please contact the corresponding author for more information.

## Declaration of interests

SC, MIP, LL, AIL, LZ, IE, TJ, LH, DF, MvF, BdP, CAJMG, HK, PPMZ, MO, PAFMD, JPGS, YD: None. LWvL: Outside the current work: Consultancy fees to UMCU from Abbott, Medtronic, Vifor, Novartis. Investigator-initiated study in collaboration with Roche (cTnT kits).
