# Supplementary material for: The circadian clock remains intact, but with dampened hormonal output in heart failure
Source: eBioMedicine. 2023 Apr 17;91:104556. doi: 10.1016/j.ebiom.2023.104556 (PMC10131037; doi:10.1016/j.ebiom.2023.104556)
Supplement: Supplementary Tables S1 and S2 and Figs. S1–S5 [file mmc1.docx]

**The circadian clock remains intact, but with dampened hormonal output in heart failure**

*Sandra Crnko PhD, Markella I. Printezi BSc, Peter-Paul M. Zwetsloot MD PhD, Laurynas Leiteris BSc, Andrew I. Lumley MSc, Lu Zhang MSc, Isabelle Ernens PhD, Tijn P.J. Jansen MD, Lilian Homsma MD, Dries Feyen PhD, Martijn van Faassen PhD, Bastiaan C. du Pré MD PhD, Carlo A.J.M. Gaillard MD PhD, Hans Kemperman PhD, Marish I.F.J. Oerlemans MD PhD, Anne M. May PhD, Pieter A.F.M. Doevendans MD PhD, Nicolaas P.A. Zuithoff, PhD, Joost P.G. Sluijter PhD, Yvan Devaux PhD, Linda W. van Laake MD PhD*

_____

**Supplementary materials**

**Table of contents**

**Supplementary Table S1:** Primer sequences used for the quantitative real-time PCR reaction…………………2

**Supplementary Table S2:** Individual cosinor parameters for melatonin, cortisol and cTnT.................................3

**Supplementary Figure S1:** Study design overview………………………………………………….………..…6

**Supplementary Figure S2:** Echocardiographic evaluation of cardiac function in mice...………………….……7

**Supplementary Figure S3:** Fitted cosinor curves of individual subjects for melatonin, cortisol and cTnT…..…8

**Supplementary Figure S4:** Confirmation of heart failure in murine hearts…………………………………….11

**Supplementary Figure S5:** Confirmation of heart failure in zebrafish hearts………………………………….12

**Supplementary Table S1: Primer sequences used for the quantitative real-time PCR reaction.**

| ***Animal*** | ***Gene*** | ***Forward primer (5' - 3')*** | ***Reverse primer (5' - 3')*** |
| --- | --- | --- | --- |
|  | *Rplp0* | GGACCCGAGAAGACCTCCTT | GCACATCACTCAGAATTTCAATGG |
| **Mouse** | *Bmal1* | TGACCCTCATGGAAGGTTAGAA | GGACATTGCATTGCATGTTGG |
|  | *Cry2* | CCTCGTCTGTGGGCATCAA | GCTTTCTTAAGCTTGTGTCCAGATC |
|  | *Clock* | AAAGACGGCGAGAACTTGG | GGAGGCAGAAGGAGTTGGG |
|  | *Per1* | TCGAAACCAGGACACCTTCTCT | GGGCACCCCGAAACACA |
|  | *Anp* | CCTGTGTACAGTGCGGTGTC | CCTAGAAGCACTGCCGTCTC |
|  | *Bnp* | GTTCTTTTGTGAGGCCTTGG | CTGAAGGTGCTGTCCCAGAT |
|  | *eef1a1* | CTTCTCAGGCTGACTGTGC | CCGCTAGCATTACCCTCC |
|  | *arntl1b* | TTCTTTTGAGGACAGTATGGAC | TGTTCATCTTGTCTCTGCGTCT |
| **Zebrafish** | *cry2a* | GCATGACATGGCAGGACACTT | TGCGATTTGCCGTTGTAACT |
|  | *clocka* | GACCACCAACCTCAACCAGCA | TCGGCTGAGAGATCATCATGGTA |
|  | *per1a* | TGGTAAAGACCAGCGGACAG | TGCCTTTGCCAGTGCTTCTA |
|  | *anp* | GATGTACAAGCGCACACGTT | TCTGATGCCTCTTCTGTTGC |
|  | *bnp* | CAGAATCGGTTCAATGTC | TTGTGAGGTTAAATCAAGTAG |

*Anp*=Natriuretic Peptide Type A; *arntl (bmal)*=Aryl Hydrocarbon Receptor Nuclear Translocator Like; *Bmal*=Brain and Muscle ARNT-Like; *Bnp=*Brain Natriuretic Peptide; *Clock*=Circadian Locomotor Output Cycles Kaput; *Cry*=Cryptochrome Circadian Regulator; *eef1a1*=Elongation factor 1-alpha 1; *Per*=Period Circadian Regulator; *Rplp0*=Ribosomal Protein Lateral Stalk Subunit P0.

**Supplementary Table S2: Individual cosinor parameters for melatonin, cortisol and cTnT.**

|  | **Melatonin** | | | | |  | **Cortisol** | | |  | **cTnT** | | | | |  |
| --- | --- | --- | --- | --- | --- | --- | --- | --- | --- | --- | --- | --- | --- | --- | --- | --- |
|  | **Mesor**  **ln** | **Mesor** | **Ampl**  **ln** | **Ampl** | **Acro** | **Cos** | **Mesor** | **Ampl** | **Acro** | **Cos** | **Mesor ln** | **Mesor** | **Ampl ln** | **Ampl** | **Acro** | **Cos** |
| **Heart failure patients** | | | | | | | | | | | | | | | | |
| **1** | 3·1 | 22·7 | 2·1 | 8·4 | 04:57 | S | 181·0 | 189·0 | 11:38 | S | -4·6 | 0·010 | 0·05 | 1·1 | 08:28 | NS |
| **2** | 2·8 | 15·9 | 1·9 | 6·9 | 04:32 | S | 411·8 | 195·7 | 09:58 | S | -3·4 | 0·034 | 0·06 | 1·1 | 17:27 | S |
| **3** | 1·9 | 6·4 | 0·9 | 2·5 | 02:21 | S | 330·6 | 208·6 | 08:00 | S | -4·8 | 0·009 | 0·10 | 1·1 | 04:28 | S |
| **4** | 2·1 | 8·2 | 1·4 | 4·1 | 02:40 | S | 373·6 | 191·7 | 10:38 | S | -3·3 | 0·036 | 0·10 | 1·1 | 07:44 | NS |
| **5** | 2·2 | 9·2 | 1·6 | 5·1 | 02:34 | S | 277·7 | 161·5 | 10:53 | S | -2·8 | 0·058 | 0·21 | 1·2 | 02:25 | NS |
| **6** | 3·3 | 26·7 | 2·3 | 10·3 | 05:06 | S | 440·4 | 236·1 | 11:35 | S | -3·6 | 0·028 | 0·09 | 1·1 | 11:21 | S |
| **7** | 3·5 | 33·0 | 2·4 | 10·8 | 05:38 | S | 392·4 | 133·2 | 09:47 | NS | -3·2 | 0·040 | 0·10 | 1·1 | 13:07 | S |
| **8** | 5·0 | 145·0 | 1·8 | 6·2 | 05:25 | S | 83·6 | 61·8 | 09:25 | S | -4·0 | 0·018 | 0·18 | 1·2 | 17:36 | S |
| **9** | 3·4 | 30·3 | 1·9 | 6·5 | 03:05 | S | 327·7 | 199·6 | 10:06 | S | -3·8 | 0·021 | 0·12 | 1·1 | 09:15 | S |
| **10** | 3·5 | 33·1 | 2·5 | 12·7 | 04:49 | S | 208·9 | 156·2 | 09:17 | S | -3·3 | 0·036 | 0·17 | 1·2 | 10:28 | S |
| **11** | 2·1 | 8·4 | 1·5 | 4·3 | 02:31 | S | 258·1 | 201·2 | 10:11 | S | -3·0 | 0·050 | 0·09 | 1·1 | 11:00 | NS |
| **12** | 2·6 | 12·9 | 1·7 | 5·2 | 04:30 | S | 371·8 | 155·0 | 10:04 | S | -3·0 | 0·050 | 0·08 | 1·1 | 14:17 | S |
| **13** | 4·7 | 114·1 | 1·7 | 5·7 | 05:12 | S | 311·1 | 159·6 | 09:07 | S | -3·6 | 0·028 | 0·14 | 1·1 | 12:08 | S |
| **14** | 2·1 | 7·9 | 1·1 | 2·9 | 04:00 | S | 259·9 | 153·2 | 08:01 | S | -5·0 | 0·006 | 0·22 | 1·2 | 23:15 | S |
| **15** | 2·7 | 14·3 | 1·0 | 2·6 | 06:01 | S | 626·0 | 200·7 | 09:53 | S | -2·8 | 0·058 | 0·05 | 1·0 | 13:30 | NS |
| **16** | 2·7 | 15·1 | 1·6 | 5·1 | 04:16 | S | 356·1 | 147·1 | 10:31 | NS | -4·7 | 0·009 | 0·10 | 1·1 | 19:35 | NS |
| **17** | 3·5 | 33·4 | 1·9 | 6·5 | 03:30 | S | 291·1 | 142·5 | 11:32 | NS | -4·2 | 0·015 | 0·07 | 1·1 | 07:00 | NS |
| **18** | 3·0 | 19·4 | 2·4 | 11·4 | 03:46 | S | 304·0 | 219·4 | 10:56 | S | -2·3 | 0·098 | 0·14 | 1·1 | 10:33 | S |
| **19** | 4·6 | 95·8 | 2·0 | 7·1 | 02:25 | NS | 263·9 | 228·6 | 10:23 | S | -3·1 | 0·043 | 0·10 | 1·1 | 10:24 | S |
| **20** | 3·8 | 45·2 | 2·0 | 7·5 | 02:11 | S | 279·1 | 126·9 | 04:52 | NS | -4·1 | 0·017 | 0·14 | 1·2 | 07:52 | S |
| **21** | 4·4 | 82·2 | 2·2 | 8·9 | 05:41 | S | 334·2 | 94·7 | 10:32 | NS | -2·6 | 0·075 | 0·06 | 1·1 | 18:26 | S |
| **22** | 3·8 | 45·5 | 2·2 | 9·4 | 05:15 | S | 181·7 | 137·1 | 09:40 | S | -3·0 | 0·049 | 0·10 | 1·1 | 11:46 | S |
| **23** | 2·4 | 10·9 | 1·1 | 2·9 | 03:47 | S | 277·6 | 228·8 | 11:20 | S | -1·1 | 0·324 | 0·04 | 1·0 | 14:03 | S |
| **24** | NA | NA | NA | NA | NA | S | 275·6 | 129·7 | 07:57 | S | -3·7 | 0·025 | 0·23 | 1·3 | 11:57 | S |
| **25** | 1·8 | 6·0 | 0·7 | 2·0 | 03:05 | S | 259·7 | 89·1 | 08:22 | NS | -3·8 | 0·022 | 0·14 | 1·2 | 12:10 | S |
| **26** | 3·0 | 20·1 | 1·9 | 6·4 | 01:40 | S | 354·8 | 179·8 | 11:17 | S | -3·2 | 0·041 | 0·10 | 1·1 | 07:02 | S |
| **27** | 3·5 | 33·5 | 2·0 | 7·7 | 04:14 | S | 476·8 | 34·6 | 21:59 | NS | -3·9 | 0·020 | 0·09 | 1·1 | 08:59 | S |
| **28** | 2·7 | 15·1 | 1·5 | 4·3 | 03:30 | NA | 439·8 | 196·3 | 08:32 | S | -4·1 | 0·017 | 0·16 | 1·2 | 13:55 | NS |
| **29** | 1·8 | 5·8 | 0·7 | 2·1 | 02:56 | S | 251·7 | 184·5 | 08:41 | S | -4·1 | 0·017 | 0·06 | 1·1 | 09:50 | NS |
| **30** | 2·6 | 13·5 | 1·8 | 6·4 | 03:47 | S | 309·9 | 158·2 | 11:43 | S | -3·7 | 0·025 | 0·05 | 1·0 | 14:58 | S |
| **31** | 3·0 | 19·4 | 2·3 | 9·8 | 04:18 | S | 296·9 | 220·3 | 11:35 | S | -4·3 | 0·014 | 0·19 | 1·2 | 14:34 | S |
| **32** | 2·7 | 14·6 | 1·9 | 6·7 | 01:01 | S | 308·3 | 118·3 | 07:12 | NS | -4·7 | 0·009 | 0·19 | 1·2 | 14:33 | NS |
| **33** | 1·9 | 6·4 | 1·0 | 2·6 | 03:02 | S | 312·6 | 182·3 | 09:38 | S | -3·4 | 0·035 | 0·15 | 1·2 | 18:38 | S |
| **34** | 2·4 | 11·5 | 1·5 | 4·4 | 05:12 | S | 462·8 | 161·5 | 08:52 | S | -3·9 | 0·021 | 0·03 | 1·0 | 18:00 | NS |
| **35** | 4·0 | 52·5 | 2·0 | 7·2 | 03:37 | S | 419·3 | 121·0 | 06:48 | S | -3·2 | 0·040 | 0·05 | 1·0 | 10:21 | S |
| **36** | 2·6 | 13·8 | 1·4 | 3·9 | 04:40 | S | 489·9 | 269·4 | 12:17 | S | -3·2 | 0·041 | 0·04 | 1·0 | 20:48 | NS |
| **37** | 5·6 | 275·6 | 1·2 | 3·2 | 04:18 | S | 483·7 | 105·1 | 09:37 | S | -3·8 | 0·022 | 0·15 | 1·2 | 13:22 | NS |
| **38** | 3·0 | 21·1 | 1·0 | 2·8 | 01:47 | S | 213·7 | 158·2 | 09:45 | S | -4·5 | 0·011 | 0·04 | 1·0 | 08:50 | NS |
| **39** | 1·6 | 5·0 | 0·5 | 1·7 | 04:11 | NS | 249·2 | 175·3 | 09:08 | S | -4·7 | 0·009 | 0·17 | 1·2 | 14:29 | S |
| **40** | 3·7 | 39·0 | 2·3 | 10·0 | 04:28 | S | 369·5 | 227·7 | 10:48 | S | -3·9 | 0·019 | 0·06 | 1·1 | 12:33 | NS |
| **41** | NA | NA | NA | NA | NA | S | 445·1 | 174·9 | 12:35 | S | -3·8 | 0·023 | 0·08 | 1·1 | 01:16 | NS |
| **42** | 1·5 | 4·7 | 0·4 | 1·4 | 04:11 | NS | 240·8 | 150·6 | 09:33 | S | -4·3 | 0·014 | 0·08 | 1·1 | 14:25 | S |
| **43** | 3·0 | 20·9 | 2·3 | 10·5 | 04:25 | S | 317·7 | 213·7 | 10:37 | NS | -4·2 | 0·015 | 0·18 | 1·2 | 11:08 | S |
| **44** | 4·1 | 59·1 | 1·3 | 3·6 | 05:18 | S | 469·6 | 155·2 | 12:05 | S | -3·0 | 0·050 | 0·04 | 1·0 | 19:08 | NS |
| **45** | 4·8 | 121·4 | 2·1 | 8·5 | 04:02 | NA | 431·9 | 209·8 | 11:47 | S | -4·0 | 0·018 | 0·15 | 1·2 | 10:32 | S |
| **46** | 3·2 | 24·1 | 1·7 | 5·5 | 03:46 | S | 266·6 | 170·4 | 09:05 | S | -3·9 | 0·020 | 0·16 | 1·2 | 11:39 | S |
| **Controls** | | | | | | | | | | | | | | | | |
| **1** | 2·7 | 15·3 | 2·0 | 7·5 | 03:48 | NA | 246·3 | 179·5 | 10:38 | S | NA | NA | NA | NA | NA | NS |
| **2** | 2·9 | 18·5 | 1·9 | 6·9 | 01:17 | S | 170·8 | 91·0 | 07:06 | S | NA | NA | NA | NA | NA | NS |
| **3** | 2·4 | 11·0 | 1·7 | 5·3 | 03:21 | S | 244·5 | 97·4 | 08:37 | S | NA | NA | NA | NA | NA | S |
| **4** | 2·7 | 15·0 | 2·1 | 7·9 | 03:43 | S | 278·0 | 138·4 | 13:07 | NS | NA | NA | NA | NA | NA | NS |
| **5** | 2·8 | 17·0 | 2·1 | 8·4 | 04:14 | S | 268·9 | 199·4 | 09:20 | S | NA | NA | NA | NA | NA | NS |
| **6** | NA | NA | NA | NA | NA | S | 192·8 | 127·1 | 09:09 | S | NA | NA | NA | NA | NA | S |
| **7** | 3·1 | 23·0 | 2·5 | 12·2 | 04:21 | S | 219·9 | 230·9 | 10:28 | S | NA | NA | NA | NA | NA | S |
| **8** | 3·2 | 24·7 | 2·6 | 13·5 | 04:27 | S | 377·4 | 327·9 | 10:08 | S | NA | NA | NA | NA | NA | NS |
| **9** | 3·0 | 21·0 | 2·4 | 11·5 | 04:06 | S | 297·1 | 231·3 | 11:27 | S | NA | NA | NA | NA | NA | S |
| **10** | 3·9 | 48·1 | 2·4 | 11·2 | 02:50 | NS | 197·5 | 134·1 | 10:10 | S | NA | NA | NA | NA | NA | NS |
| **11** | 3·8 | 45·9 | 2·9 | 18·1 | 03:05 | S | 477·6 | 124·1 | 12:26 | S | NA | NA | NA | NA | NA | NS |
| **12** | 2·8 | 15·8 | 2·0 | 7·7 | 04:26 | S | 239·0 | 187·2 | 09:53 | S | NA | NA | NA | NA | NA | NS |
| **13** | 3·0 | 20·1 | 2·4 | 10·8 | 03:51 | S | 240·8 | 145·0 | 08:50 | S | NA | NA | NA | NA | NA | NS |
| **14** | 2·9 | 17·8 | 2·3 | 9·8 | 04:45 | S | 282·7 | 205·0 | 09:10 | S | NA | NA | NA | NA | NA | S |
| **15** | 3·3 | 28·5 | 2·4 | 10·9 | 05:20 | S | 236·0 | 191·3 | 10:51 | S | NA | NA | NA | NA | NA | S |
| **16** | 2·3 | 10·0 | 1·7 | 5·2 | 03:24 | S | 325·5 | 227·5 | 09:44 | S | NA | NA | NA | NA | NA | S |
| **17** | 3·3 | 28·3 | 2·5 | 12·4 | 03:32 | S | 374·2 | 155·8 | 05:20 | NS | NA | NA | NA | NA | NA | S |
| **18** | 2·5 | 12·4 | 1·6 | 5·0 | 04:38 | S | 299·7 | 176·4 | 08:22 | S | NA | NA | NA | NA | NA | NS |
| **19** | 2·4 | 10·9 | 0·9 | 2·6 | 04:15 | S | 220·3 | 160·4 | 09:57 | S | NA | NA | NA | NA | NA | NS |
| **20** | 3·2 | 25·8 | 2·6 | 13·8 | 04:24 | S | 318·1 | 135·9 | 12:20 | S | NA | NA | NA | NA | NA | S |
| **21** | 2·6 | 12·9 | 1·7 | 5·6 | 03:55 | S | 225·3 | 133·5 | 10:33 | S | NA | NA | NA | NA | NA | S |
| **22** | 3·8 | 43·7 | 2·2 | 9·0 | 03:24 | S | 280·8 | 206·3 | 09:58 | S | NA | NA | NA | NA | NA | NS |
| **23** | 2·8 | 17·1 | 2·2 | 9·1 | 01:18 | S | 273·1 | 229·0 | 10:49 | S | NA | NA | NA | NA | NA | S |
| **24** | 2·8 | 15·8 | 2·1 | 8·0 | 03:42 | S | 368·1 | 71·2 | 04:26 | NS | NA | NA | NA | NA | NA | S |

Acro=acrophase; ampl=amplitude; Cos= cosinor fit, cTnT=cardiac troponin T; ln=log transformed; NA=not applicable; NS= not significant; S= significant.

**
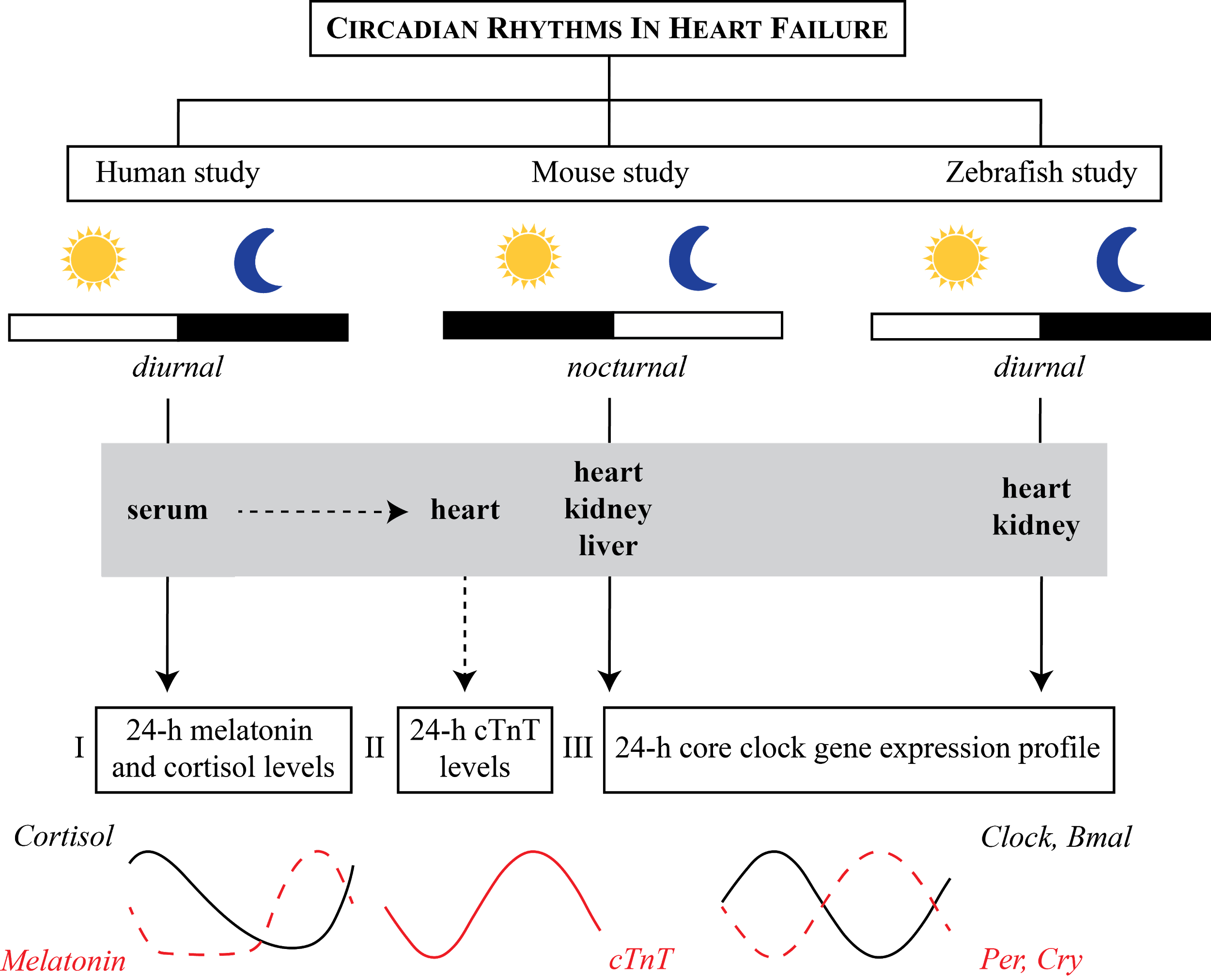
**

**Supplementary Figure S1: Study design overview.** In order to characterise circadian rhythmicity in heart failure patients, three separate studies were performed: human, mouse, and zebrafish study. 24-hour serum samples were collected from heart failure patients and healthy controls to assess the rhythmic expression of main endocrine products of the central clock: melatonin and cortisol. Furthermore, since human hearts are not accessible for repeated sampling, serum cardiac troponin T was used as a surrogate clock marker reflecting cardiac-specific rhythms. The functionality of the peripheral circadian clock was further analysed in the hearts of nocturnal mice (opposite rest/activity cycles to humans) and diurnal zebrafish (the same rest/activity cycles as humans) with heart failure, as well as in their respective controls without heart failure. Finally, circadian rhythmicity was assessed in other target organs in heart failure: kidneys and liver. *Bmal*=Brain and Muscle ARNT-Like; *Clock*=Circadian Locomotor Output Cycles Kaput; *Cry*=Cryptochrome Circadian Regulator; cTnT=cardiac troponin T; *Per*=Period Circadian Regulator.

**
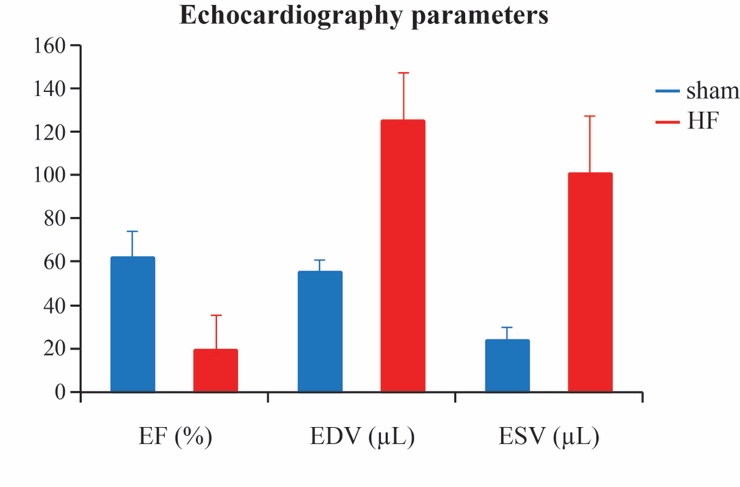
**

**Supplementary Figure S2: Echocardiographic evaluation of cardiac function in mice.** EDV=end diastolic volume; EF=ejection fraction; ESV=end systolic volume; HF=heart failure.

**Melatonin**

**
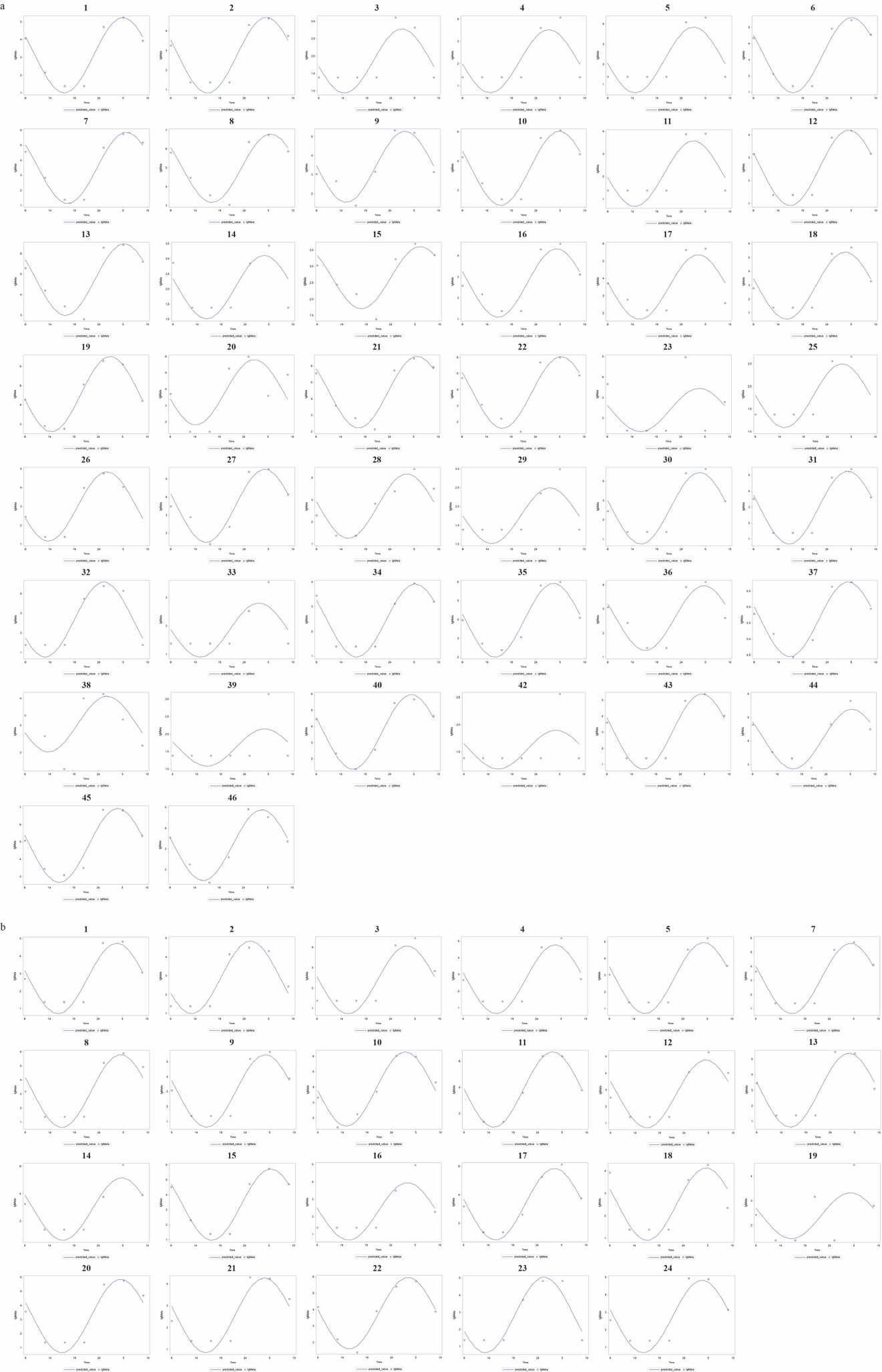
**

**Cortisol**

**
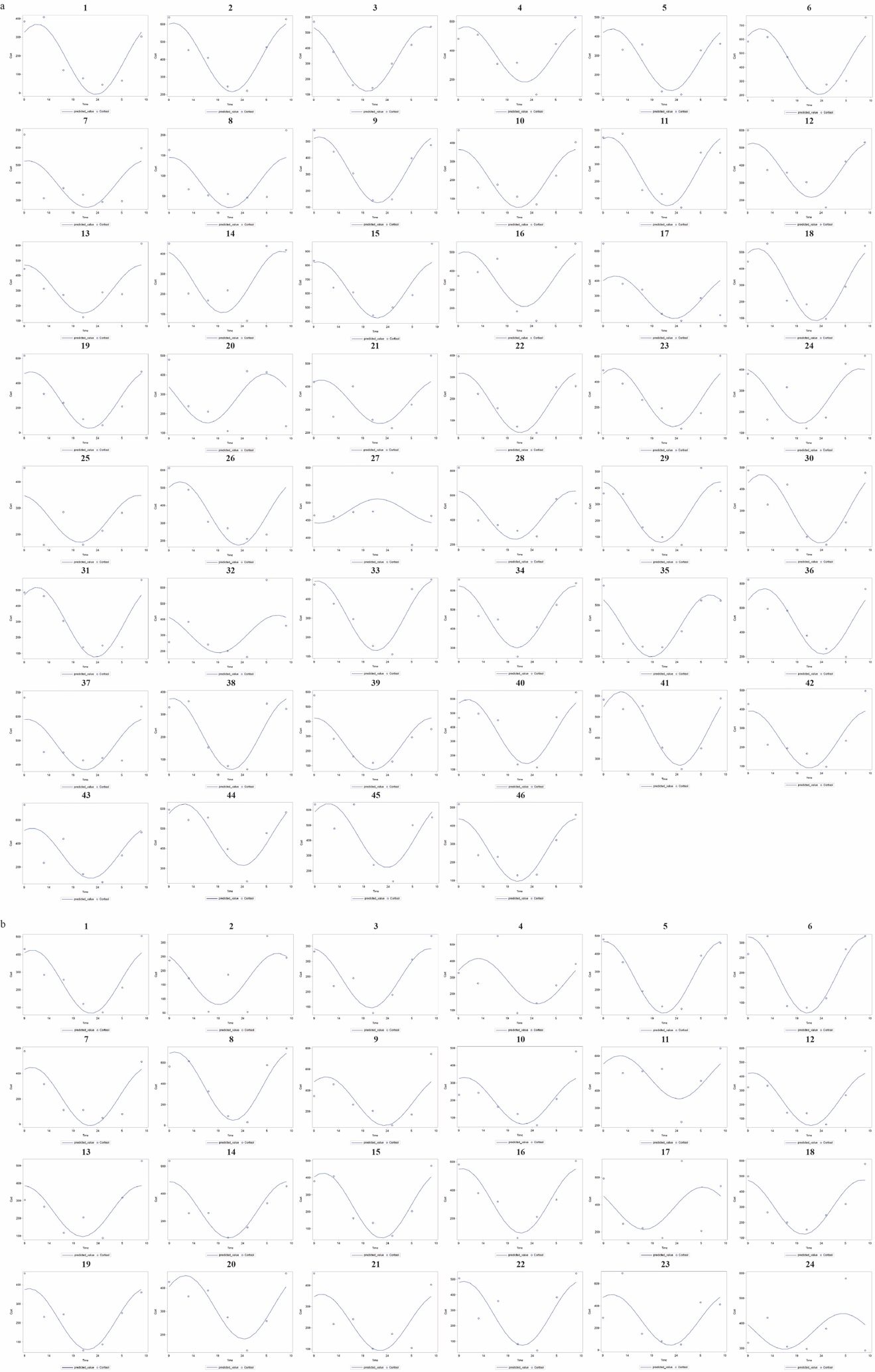
**

**cTnT**


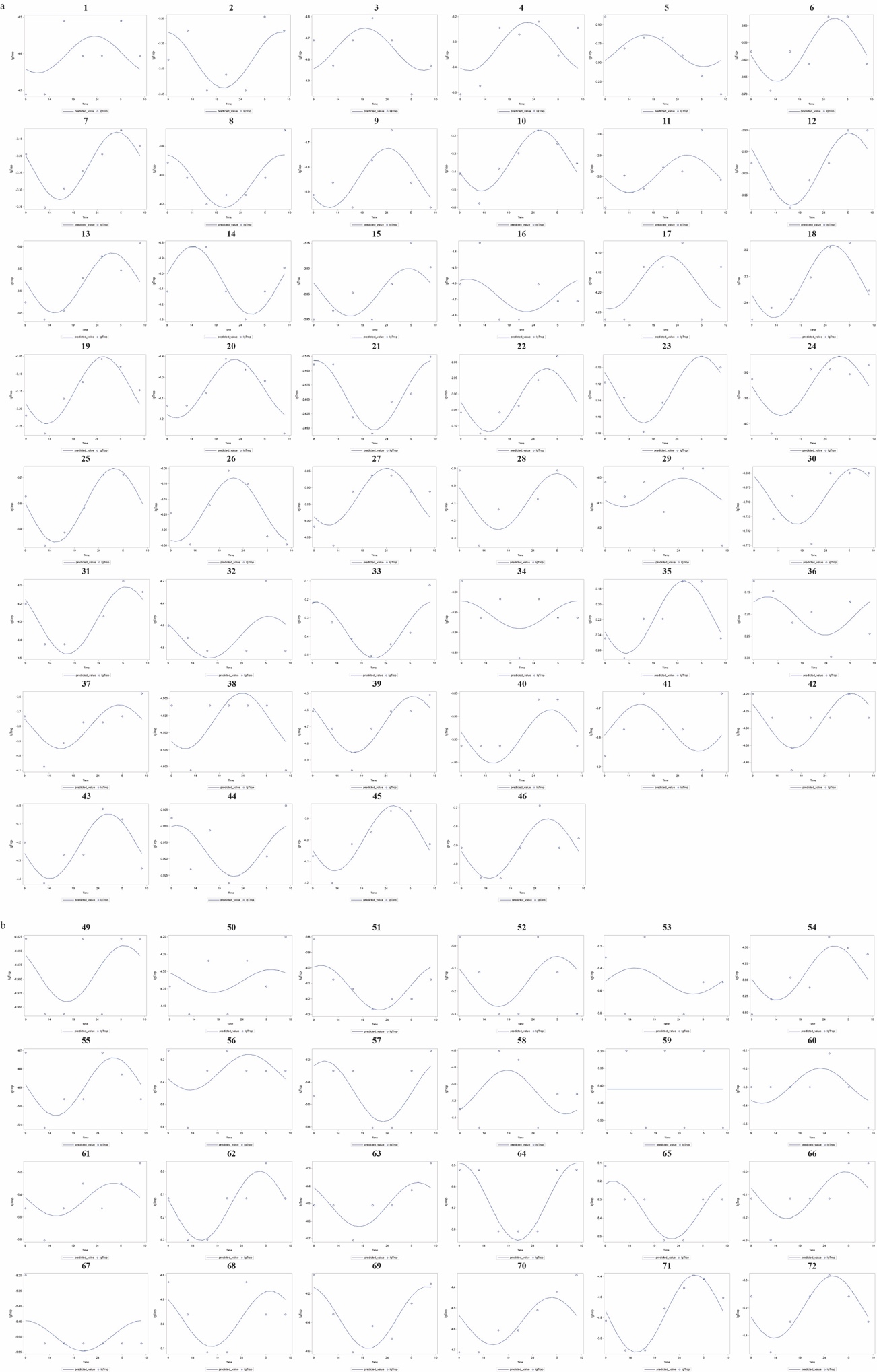


**Supplementary Figure S3: Fitted cosinor curves of individual (a) patients and (b) controls for melatonin, cortisol and cTnT.** Melatonin and cTnT were log transformed in order to achieve approximate normality. cTnT= cardiac troponin T.


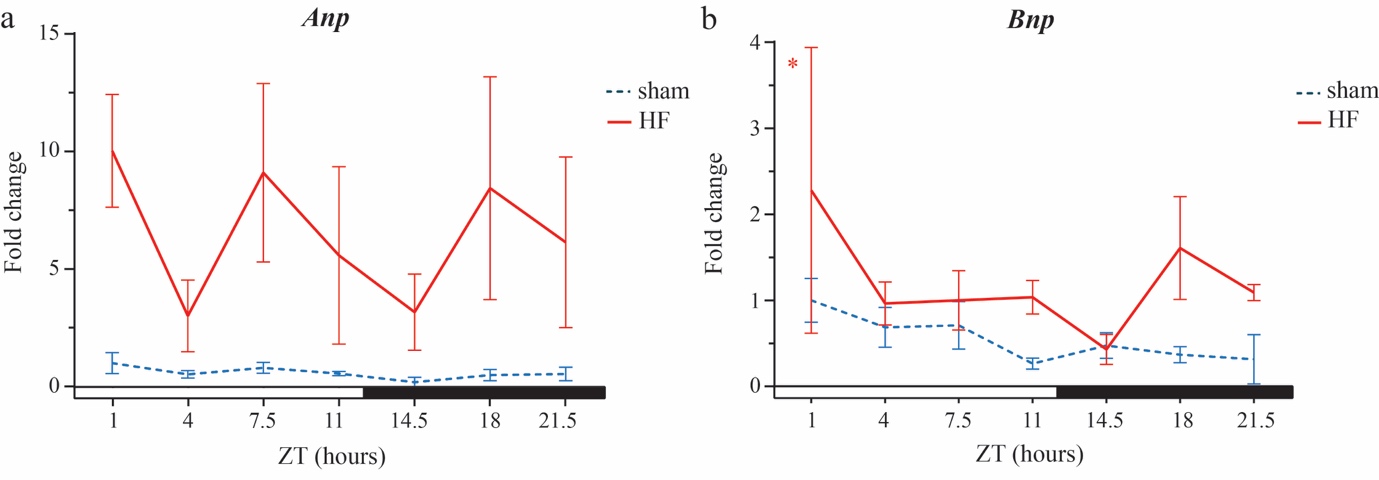


**Supplementary Figure S4: Confirmation of heart failure in murine hearts.** Comparison of 24-hour **(a)** *Anp* and **(b)** *Bnp* expression levels in murine heart between sham control and HF group, as determined by quantitative real-time PCR reaction (N=3-5 mice/group/ZT). Values are mean ± SEM. Data are normalised against ZT1 of the sham control group. *P*<0·05 was used as a cut-off for significance. Statistical significance of cosinor rhythmicity of dCt values per group is indicated at the top left graph corner: **P*<0·05. Horizontal bar indicates lights-on (=white; ZT0) and lights-off (=black; ZT12) period. *Anp*=Natriuretic Peptide Type A; *Bnp*=Brain Natriuretic Peptide; HF=heart failure; ZT=zeitgeber time.

**
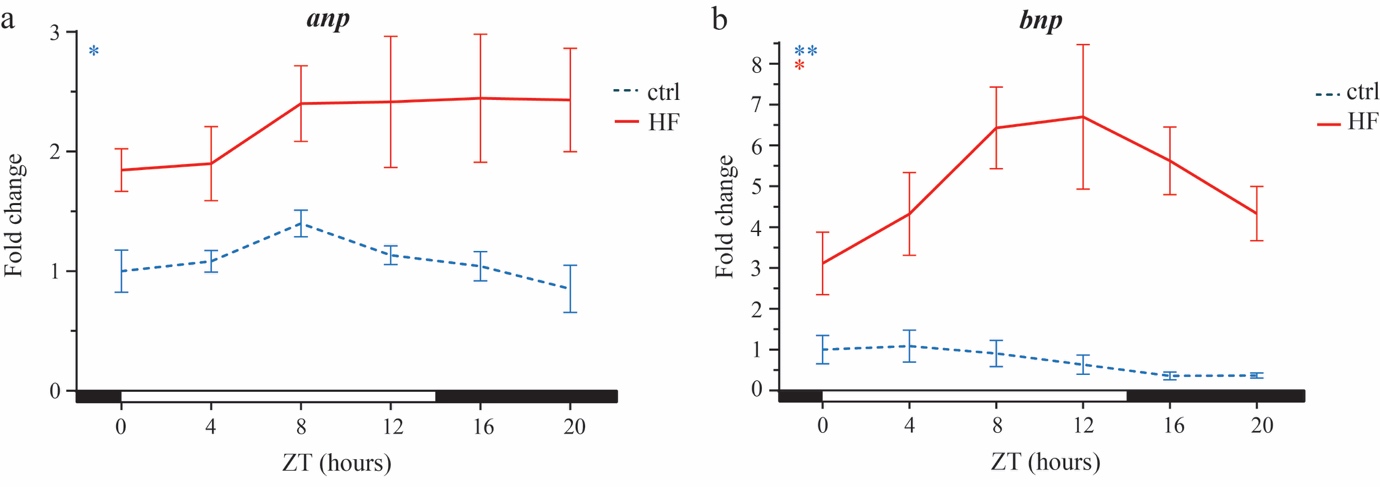
**

**Supplementary Figure S5: Confirmation of heart failure in zebrafish hearts.** Comparison of 24-hour **(a)** *anp* and **(b)** *bnp* expression levels in zebrafish heart between control and HF group, as determined by quantitative real-time PCR reaction (**(a)** N=6 and **(b)** N=9 zebrafish/group/ZT; 2 and 3 biological replicates each including 3 pooled ventricles, respectively). Values are mean ± SEM. Data are normalised against ZT0 of the control group. *P*<0·05 was used as a cut-off for significance. Statistical significance of cosinor rhythmicity of dCt values per group is indicated at the top left graph corner: **P*<0·05, ***P*<0·01. Horizontal bar indicates lights-on (=white; ZT0) and lights-off (=black; ZT14) period. *anp*=Natriuretic Peptide Type A; *bnp*=Brain Natriuretic Peptide; HF=heart failure; ZT=zeitgeber time.
